# The research on the particle concentration distribution of directed airflow in cleanrooms for operators

**DOI:** 10.1371/journal.pone.0296803

**Published:** 2024-03-01

**Authors:** Chao Li, Hao Li, Minwei Zhang, Xin Wang, Cui Huang

**Affiliations:** 1 School of Environment and Architecture, University of Shanghai for Science and Technology, Shanghai, China; 2 City University of Hefei, Hefei, China; Vellore Institute of Technology, INDIA

## Abstract

Existing research of non-unidirectional cleanrooms generally suggests that lower-side return air outlets provide better control effect on indoor particle concentration. As a result, there has been relatively less focus on return air outlets. However, installing return air outlets oriented towards operators as particle emission sources can reduce the impact on process layout and improve space utilization, while also provide less impact from upper particle emission sources on the workbench area. To investigate the characteristics of return air outlet for operators (abbreviated as ***H***), this study compared the particle concentration distribution, non—uniformity, and purification efficiency of return air oultet ***H*** and the traditional lower-side (abbreviated as ***L***) return air outlets by experiments and CFD simulations. Based on the theory of mass conservation, the expression of required air supply volume under equivalent cleanroom conditions was derived. Under corresponding experimental and simulation conditions, the particle concentration differences range from 2.0% to 12.7% for return air outlet ***H*** and from 12.4% to 33.2% for return air outlet ***L***, and these differences gradually decrease with the air exchange rate (ACH) increases. The results show that ACH = 20 is sufficient for cleanliness requirements with return air outlet ***H*** when there is one person in the cleanroom, while a higher rate of ACH = 35 is needed when there are two persons. Although lower-side return air outlets have certain potential for reducing particle concentration in the cleanroom, increasing the air exchange rate remains the most effective method to control indoor particle concentration. Compared to the traditional lower-side return air outlet ***L***, the ranges of the non-uniformity coefficients for return air outlet ***H*** and ***L*** are 0.50 to 0.67 and 0.45 to 0.53, respectively. The average non-uniformity coefficient differs by 11.9%, and there is not a significant difference in uniformity with more than 20 air changes per hour. The use of return air outlets ***H*** only requires an additional 11% of air supply volume to achieve the same cleanliness, demonstrating its effectiveness in controlling particle concentration. It is suitable for cleanrooms with higher requirements for workbenches and for cleanrooms with restricted floor usage or requiring flexible layouts. The study also explores the impact of width of return air outlet oriented towards operators as particle emission sources, the results show that the larger-sized outlets facilitate the particle discharge and control the particle distribution inside the room.

## 1 Introduction

With the vigorous development of global society, economy, and industrial technology, the cleanroom industry continues to progress [1, 2]. A cleanroom is a controlled environment with strict requirements for temperature, humidity, pressure, noise, and cleanliness. Its main function is to control indoor pollutants precisely, and cleanliness is an important indicator reflecting the degree of indoor contamination [3]. Cleanliness is influenced by numerous factors, including air filtration systems, human activities, indoor equipment, and airflow pattern performance [4–8]. The airflow pattern has a significant impact on cleanliness and pollution control in cleanrooms; proper airflow pattern ensures uniform air movement and prevents the accumulation of contaminants. The airflow pattern in cleanrooms is classified into two main forms: non-unidirectional and unidirectional flow. Unidirectional airflow creates a uniformly distributed air stream. For example, laminar airflow systems are effective in reducing infection risks during contaminated surgical procedures [9]. Non-unidirectional airflow cleanrooms have complex and varied airflow patterns, and are widely used [10–12]. This study selects a non-unidirectional airflow cleanroom for research.

The distribution of particles in a non-unidirectional airflow cleanroom is closely correlated with factors such as the layout of supply and return air outlets, indoor particle emission, ACH, and other factors [13–15]. The current studies on the particle concentration distribution factors mostly focuses on the structure and positioning variations of the air supply outlets [16–20]. Compared with supply air, according to the fluid dynamics theory, the "Confluence" at the return air outlet has a smaller impact on the indoor airflow. However, its effect on the airflow pattern in cleanrooms should not be ignored. Although research on the size of return air outlets still appears limited, the effect on their location has received a degree of attention. Particles generated near the return air outlet can be more effectively expelled [21]. Regarding the research on different forms of return air systems in non-unidirectional airflow cleanrooms, Grau-Bové [22] mentioned that the arrangement of the return air outlets directly affects cleanliness. At present, the most common practice for return air outlets is top-supply side-return, which has better inductive effect on particle discharge [23, 24].

Most of the previous studies on return air outlets aimed at the location and number [25–27], while analyses of different heights of return air outlet locations on the stability and efficiency of indoor airflow are relatively lacking. It is because that the lower-side was commonly regarded as effective in the concept of return air design [28–30]. ASHRAE Standard 170–2017 [31] recommends the adoption of lower-side return airflow in the design of hospital surgical cleanrooms. However, the lower-side return air inevitably affected by the impact of process equipment and personnel, lead to particles not promptly discharged; if the lower-side return air outlet to ensure the efficiency of the low-side, requires the sacrifice of the utilization of the low side of the space. Lower-side return air also caused particles disturbed around the floor and re-enter the clean zone. Return air outlets are more likely to attract additional contaminants such as hair and textile fibers, causing contamination of the filtration system and increasing maintenance costs [32]. In some cases, the airflow path of the higher-side return air outlet may differ from the lower-side return air outlet, leading to incomplete mixing of clean air. However, for complex cleanrooms with a large amount of equipment, installing higher-side return air outlets provides greater flexibility in indoor equipment layout. In operating rooms, higher-side return air outlets can reduce the concentration of contaminants at the working level, mitigate the impact of surgical smoke on the surgical area, and enhance the protection of the surgical site [33].

In short, under the assurance of a certain cleanliness, the return air outlet oriented towards operators as particle emission sources provides a solution to the problem of underutilization of the bottom space, and has less impact on particles on the floor and in the vicinity. But there is a lack of deep research, whose effects and applications are poorly defined. The location of the return air outlet affects the performance of the ventilation system [34]. Ren [35] evaluated the particle concentration decay rate and exposure risk to medical staff at different heights of return air outlets. They found that under top-supply side-return, the most effective return air height is not necessarily the lower side. Wang [36] analyzed the control effect of pollutant removal with different numbers of return air outlets in non-unidirectional flow cleanrooms and found that, under suitable conditions, increasing return air area resulted in higher expulsion efficiency and better airflow velocity distribution. These studies demonstrate the significant influence of different return/exhaust air airflow patterns on the clean environment. Additionally, minimizing the interference of equipment layouts on the airflow path and allowing it to pass through the clean zone as much as possible can achieve better results [37].

The purpose of this study was to explore the directional airflow characteristics of return air outlets for operator and establish its practical value by comparing it with conventional lower-side return air outlet. To achieve the objective, firstly, two typical heights and variations in the size of return air outlets effect on the indoor particle uniformity and purification capability were examined in a laboratory. Next, the further air changes and particle emissions were simulated by CFD. Additionally, based on the law of conservation of mass, the suitability was determined by analyzing the difference in required air supply volume for achieving the same effect.

## 2 Methods

### 2.1 Laboratory and measurement methods

**Fig 1:**
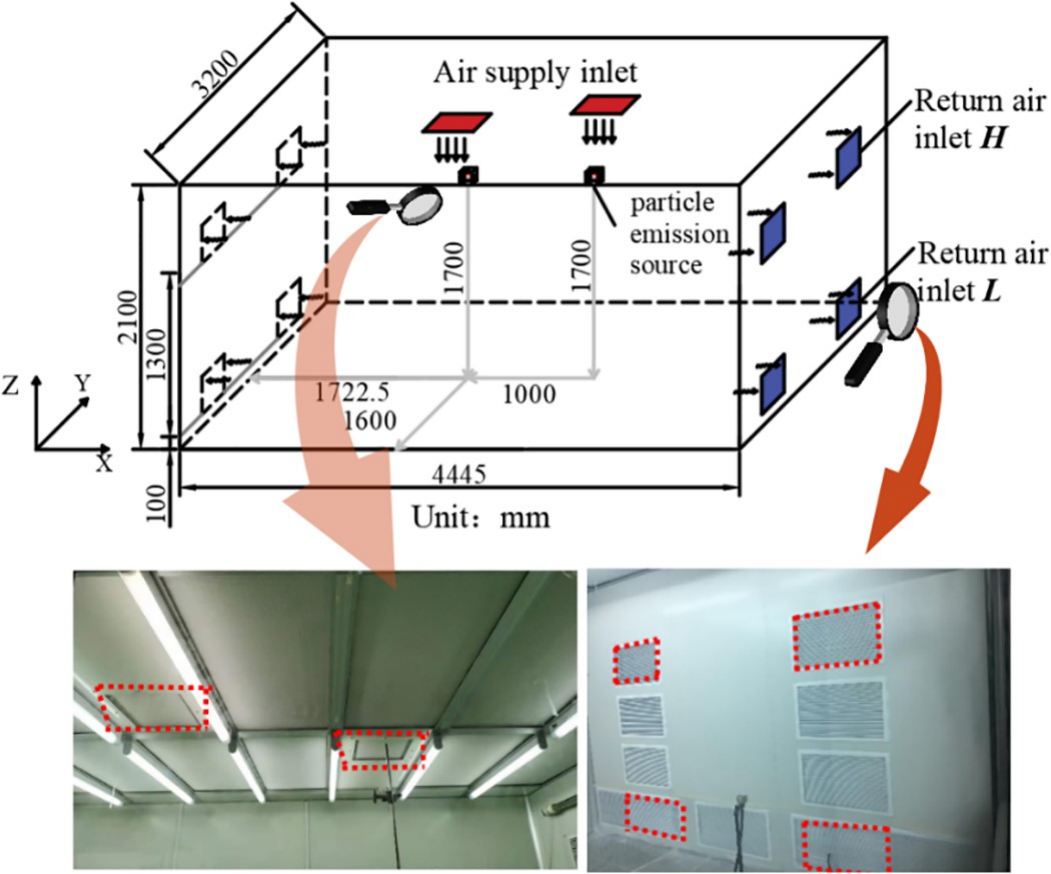
Top supply and side return clean laboratory and layout of experimental air vents.

The geometric dimensions of the experimental cleanroom are 4445 mm × 3200 mm × 2100 mm (29.8 m^3^). The ceiling of the laboratory is equipped with two FFUs, with supply air outlet sizes of 380 mm × 380 mm, as shown in Fig 1. The return air in the laboratory is arranged symmetrically on the east and west sides, and return air outlet installations have two different heights. The lower edge of the return air outlet installed on the side wall is 100 mm above the ground, referred to as the "Low" height of the return air outlet (***L***). The lower edge of the return air outlet installed on the side wall is 1300 mm above the ground, and the top height is 1600mm, which is close to the height of the personnel emission source, referred to as the "High" height of the return air outlet (***H***).

**Fig 2:**
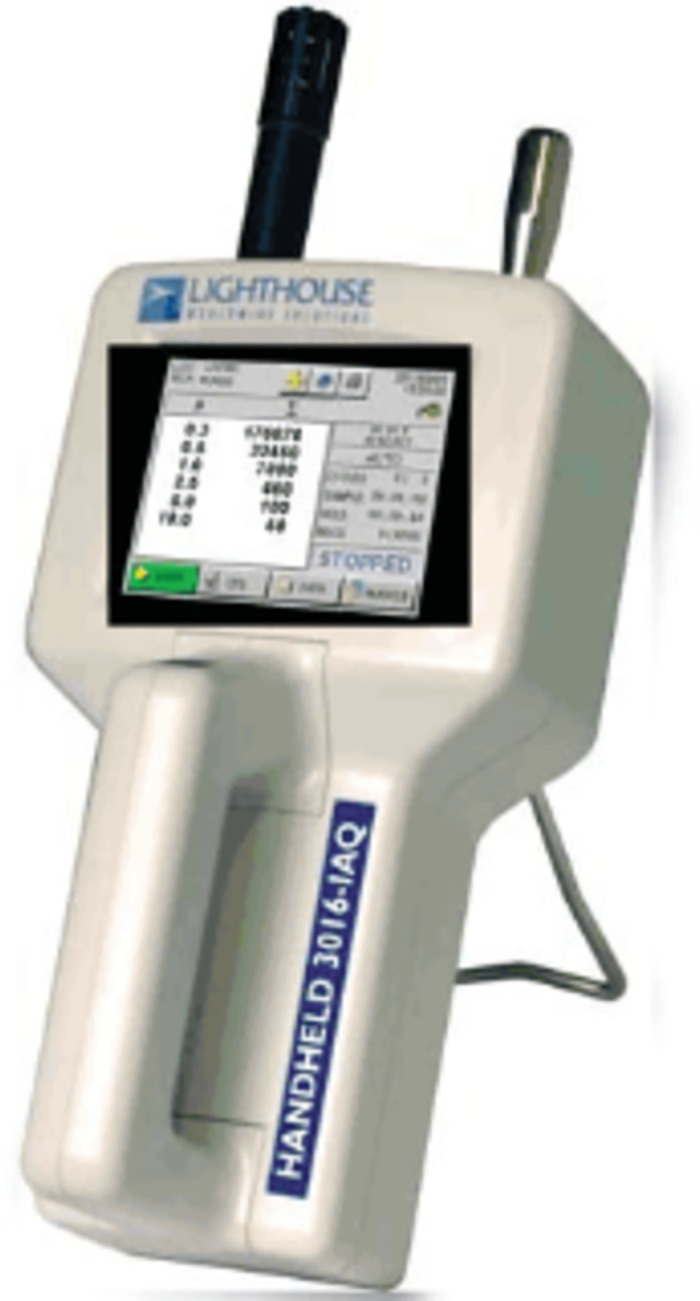
Layout of plane measurement points at three different heights and the vicinity of the source area.

**Fig 3:**
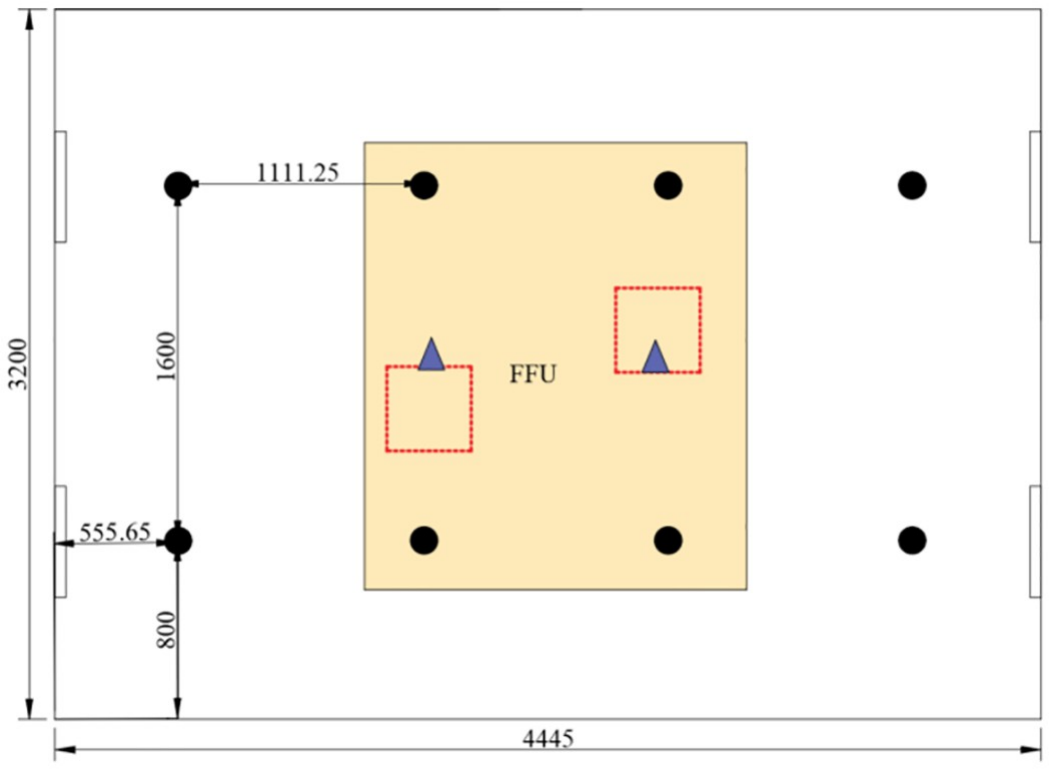
Main testing instruments for the experiment.

The indoor and source particle concentrations are measured using the Lighthouse 3016 and GRIMM Spectrometer, whereas the air volume and air velocity are measured using a matrix anemometer and an ultrasound anemometer. Fig 2 presents the main testing instruments for the experiment. The area of the cleanroom in this experiment is approximately 14.3 m^2^. According to the cleanroom testing specifications in ISO 14644 [38], a minimum of six measurement points should be used. In this experiment, eight measurement points are evenly distributed throughout the cleanroom, as shown in Fig 3. ISO 14644–3 specifies the conventional measurement height at plane of work activity. To analyze the distribution of particle concentration in the clean laboratory space comprehensively, three measurement heights are set in this study: at 0.8 m (workbench height), 1.2 m (breathing zone height when sitting), and 1.5 m (breathing zone height when standing).

### 2.2 Experimental parameters and operating conditions

Two FFUs are chosen for the experiment, with a single unit air volume of 164 m^3^/h, approximately equivalent to an air change rate of 11 times/h. The parameters varied during the experiment include the indoor unit volume particle emission rate, return air outlet height, and return air outlet size. Human activities in the cleanroom can result in particle emissions, and the number of occupants somewhat determines the indoor particle emission rate. In this study, the research includes the evaluation of unit volume particle emission rates, considering the influence of occupants. The recommended particle emission rate [39] is used to calculate the unit volume particle emission rate per person, which is approximately 33500 pc/(m^3^·min). For the experimental design, three different unit volume particle emission rates are selected: one person at 33,500p c/(m^3^·min), two persons at 67,000 pc/(m^3^·min), and five persons at 167,500 pc/(m^3^·min), which may slightly differ from the measured average unit volume particle emission rates. During the experiment, the particle concentration is monitored using the GRIMM Spectrometer particle counter. Polystyrene microspheres are used as the particle emission source with a particle diameter of 0.3 μm and a density of 1050 kg/m^3^. Two particle emission sources are symmetrically arranged, as shown in Fig 1, to achieve uniform dispersion of particles from the emission sources in the cleanroom.

As shown in Table 1, eight experimental scenarios were designed for this study. Cases 1 to 6 in the experimental conditions compare two different return air outlet heights under varying particle emission rates. Cases 6 to 8 examine the effects of changing the width of the return air outlet.

**Table 1:** Experimental conditions of return air outlet at different heights and sizes.

Experiment number	ACH	Particle emission (pc/(m^3^·min))	Size of return air outlet (mm×mm)	Height of return air outlet
Case-1	11	36560	500×300	***L***
Case-2	11	36560	500×300	***H***
Case-3	11	70500	500×300	***L***
Case-4	11	70500	500×300	***H***
Case-5	11	165160	500×300	***L***
Case-6	11	165160	500×300	***H***
Case-7	11	165160	300×300	***H***
Case-8	11	165160	100×300	***H***

### 2.3 Evaluation indicators for particle concentration

The following metrics were selected to analyze the distribution of indoor particle concentration quantitatively:

(1) Calculation formula of concentration to dust ratio
  The larger the particle generation emission, the higher the average indoor particle concentration. The concentration ratio *g* (ratio of the average indoor particle concentration to the particle emission rate) is used to reflect the contribution of airflow organization formed by different return air outlets in a cleanroom to the air purification effectiveness. A smaller concentration ratio indicates a stronger purification capability of the cleanroom. The formula is as follows:
$$g=\frac{\bar{C}}{G},$$ (1)
  where $\bar{C}$ *is* the average indoor particle concentration, (pc/m^3^) and *G* is the particle emission, (pc/(m^3^·min)).
(2) Definition and calculation formula of non-uniformity coefficient
  The uniformity reflects the distribution of particle concentrations within a space. The non-uniformity coefficient Kc is used to describe the uniformity of particle concentration distribution in the indoor environment. A higher value of *K*_*c*_ indicates a greater degree of non-uniformity in the distribution of indoor particle concentrations. The calculation formula for Kc is given by Eq (2).
$$K_{c}=\frac{1}{\bar{C}}\sqrt{\frac{\sum_{i=1}^{n}{\left(C_{i}-\bar{C}\right)}^{2}}{n}},$$ (2)
  where $\bar{C}$ is the average particle concentration values of *n* indoor measurement points, (pc/m^3^); *C*_*i*_ is the particle concentration value of the *i-th* measuring point in the experiment, (pc/m^3^); and *n* is number of measuring points in the clean room.
(3) Calculation of indoor particle concentration based on uniform distribution theory
  In the theoretical research on cleanrooms, Zhou [40] discusses four models for calculating particle concentration and proposes an improved model that can be referenced. Zhao [41] derives a theoretical expression for clean air volume based on existing expressions for non-uniform environmental concentration [An analytical expression for the transient distribution of passive contaminant under a steady flow field]. Zhao points out significant differences between the theoretical calculations using the uniform method and the non-uniform method.
  According to the theory of uniform distribution [39] in a cleanroom under mass conservation, the transient calculation equation can be derived. When the cleanroom’s air supply is in a steady state, the indoor stable particle concentration ***N*** is related to the particle emission rate and the air change rate. In other words, *N* = *f*(*G*/*n*). After derivation, the equation for indoor particle concentration is as follows:
$$N=\frac{60G\times 10^{-3}}{n\times \eta_{r}},$$ (3)
  where *N is* the stable particle concentration, (pc/L); *G* is the indoor particle emission, (pc/(m^3^•min)); *n* is air changes rate; and *η*_r_ is the total efficiency of the filter on the return air path.

### 2.4 Numerical simulation

CFD numerical simulation techniques can provide quantitative evaluations of airflow parameters in indoor environments, and their feasibility for various airflow organization studies has been widely recognized. This study used CFD to analyze the distribution of particle concentration at two typical heights to calculate the differences in airflow under change arrangements.

A cleanroom was constructed by the experimental geometry, as depicted in Fig 1. During the simulation process, to ensure consistency between the simulated particle mass and the experimental conditions, the same particle material used in the experiment was selected, and the particle density in the simulation was set to 1050 kg/m^3. A spherical particle with a radius of 0.02 m was chosen as the indoor pollutant emission source. The dimensions of the FFU air supply outlet were 380 mm × 380 mm, whereas the dimensions of the return air outlet were 500 mm × 300 mm. The location of the indoor pollutant emission source in the simulation was the same as in the experiment.

**Fig 4:**
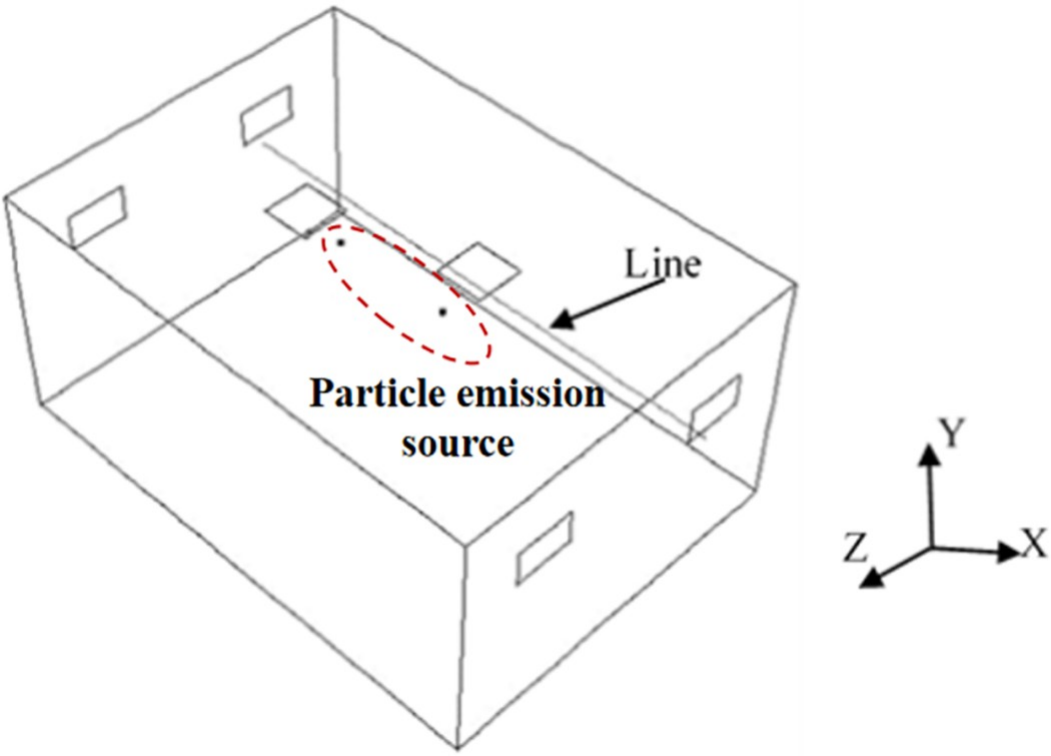
Layout of typical monitoring line and particle emission source.

The simulation incorporates the Boussinesq density assumption to consider the indoor buoyancy force, whereas the standard wall function approach is used for the wall zone. The simulation of particle concentration distribution employs the Lagrangian Discrete Phase Model method to track particle trajectories. During the simulation, the air supply velocity of a single FFU is set to 0.58 m/s, consistent with the experiment, corresponding to an air exchange rate of approximately 11 times per hour. Typical measurement lines of airflow velocity shown in Fig 4 are selected for comparison between experimental and simulated results to validate the simulation results. The measurement line is positioned at a height of 1.2 m below the air supply outlet. Ten measurement points are placed along the measurement line, and ultrasonic anemometers with a measurement range of 0–10 m/s and an accuracy of ±0.02 m/s are used to measure the airflow velocity for 6 minutes. The average value of the measured data is taken as the experimental result.

**Fig 5:**
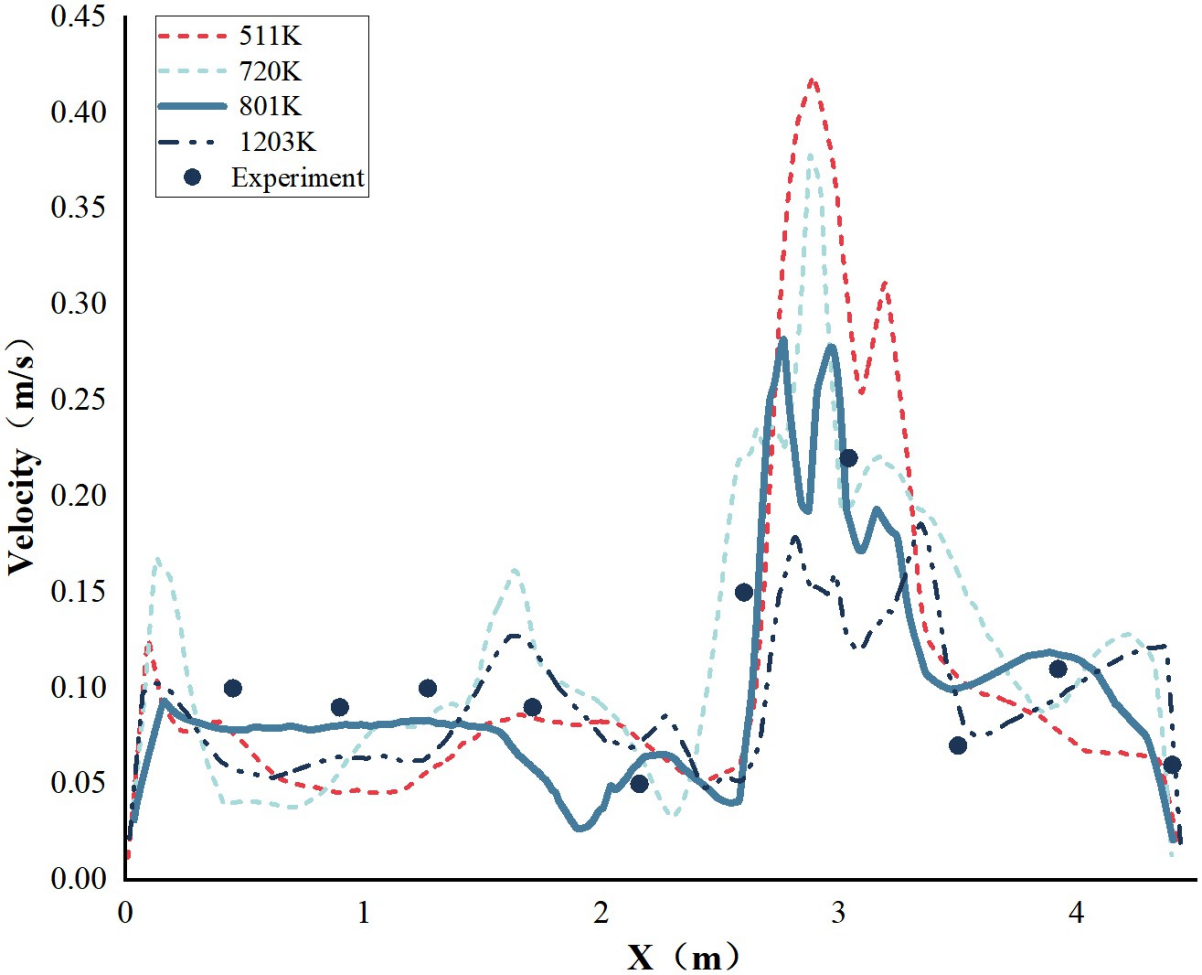
Comparison of velocity profile on monitoring line with different grid resolutions.

Four different grid resolutions were employed to examine the independence of the grid. The Fig 5 displays a comparison between the simulated and experimental velocity values at the typical measurement line for each grid resolution. With grid resolutions of 511,000 and 720,000 cells, the simulated velocity distributions deviate significantly from the experimental trends. However, with grid resolutions of 801,000 and 1,200,000 cells, the simulated velocity distributions closely match the experimental trends. Considering the computational time, a grid resolution of 801,000 cells is selected for the simulation.

Based on different air changes and particle emission, nine sets of numerical simulation conditions were set, each containing two heights, as shown in Table 2.

**Table 2:** Numerical conditions.

Numerical conditions	Height of return air outlet	*ACH* /h	particle emission (pc/(m^3^·min))
1	***H/L***	20	5000
2	***H/L***	20	15000
3	***H/L***	20	36560
4	***H/L***	20	70500
5	***H/L***	35	5000
6	***H/L***	35	15000
7	***H/L***	35	36560
8	***H/L***	35	70500
9	***H/L***	15	36560
10	***H/L***	25	36560
11	***H/L***	30	36560

### 2.5 Model validation

**Fig 6:**
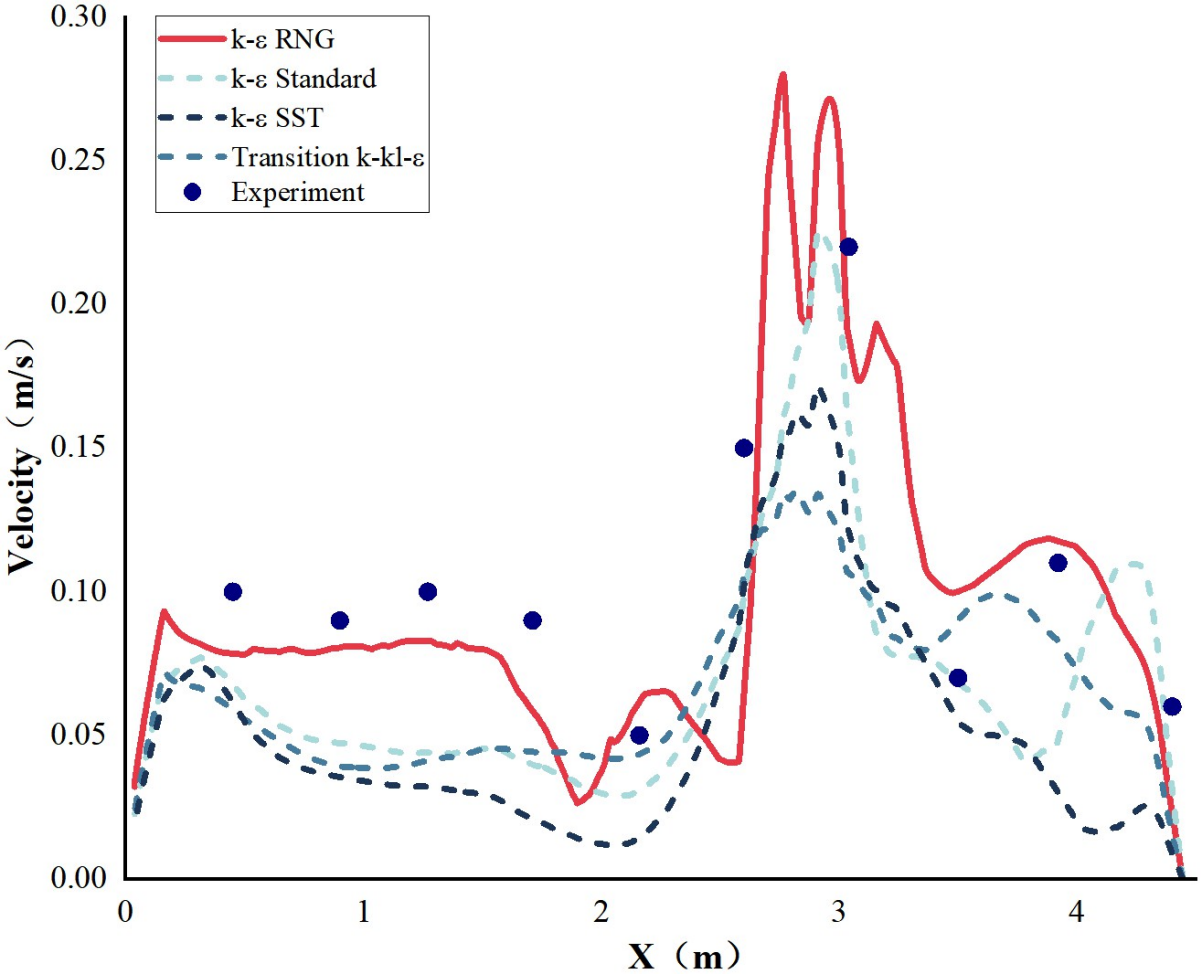
Comparison and validation of velocity profile on monitoring line with different turbulence models.

In predicting the airflow field accurately, selecting an appropriate turbulence model is crucial because the airflow in cleanrooms is predominantly turbulent. Currently, Reynolds-Averaged Navier-Stokes simulation with various turbulence models is widely used. The commonly used turbulence models include k-ε RNG, k-ω SST, Transition k-kL-ε, and k-ε Standard. In Fig 6 and S1 Table, the airflow velocity distributions for the four models were compared along a typical measuring line (Line). The results show that the k-ε RNG model predicts the indoor airflow velocity in the cleanroom most accurately, with the smallest average deviation from the measured values among the 10 monitoring points, with a maximum deviation of 0.03 m/s. Based on this comparison, the k-ε RNG model is selected for this study.

## 3 Results

### 3.1 Experimental results

#### 3.1.1 Influence of the height change of the return air outlet on the particle distribution

In cleanrooms, larger return air outlet sizes are commonly used because they cover a wider area, resulting in less disruption to particle discharge due to the larger area. Therefore, this study utilizes a large return air outlet size of 500 mm × 300 mm for preliminary analysis.

**Fig 7:**
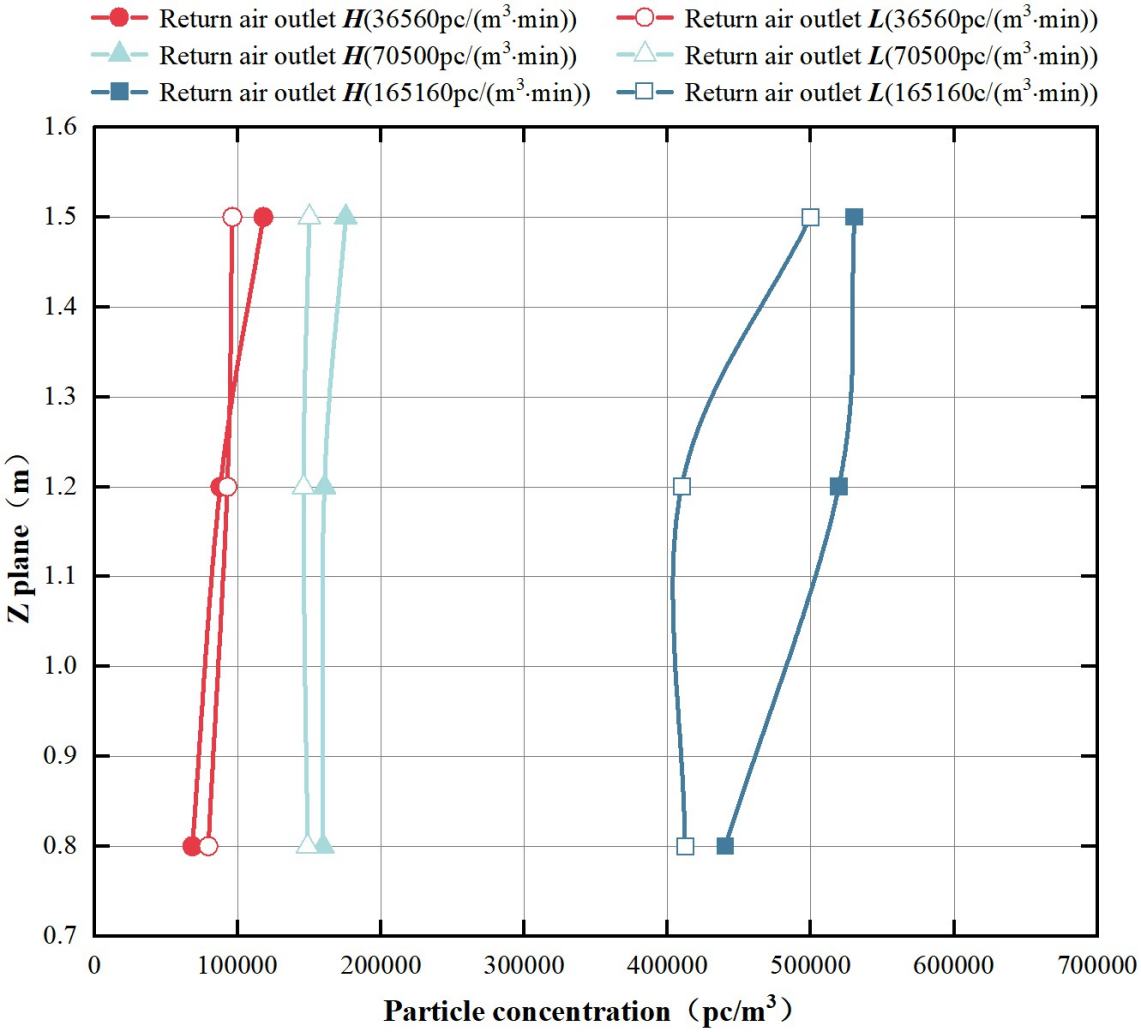
Vertical concentration at different return air outlet heights.

Fig 7 presents the concentration distribution in the vertical direction at different heights of the return air outlet. Regardless of the two different return air outlet heights, the overall variation of indoor particle concentration in the vertical direction is similar. As the height increases, the concentration slightly increases because of the position of the release source. At the return air outlet ***H***, the particle concentration at the three measurement heights is higher than at the return air outlet ***L*** for particle emission rates of 70500 pc/(m^3^·min) and 165160 pc/(m^3^·min), with the most significant difference observed at 165160 pc/(m^3^·min).

**Fig 8:**
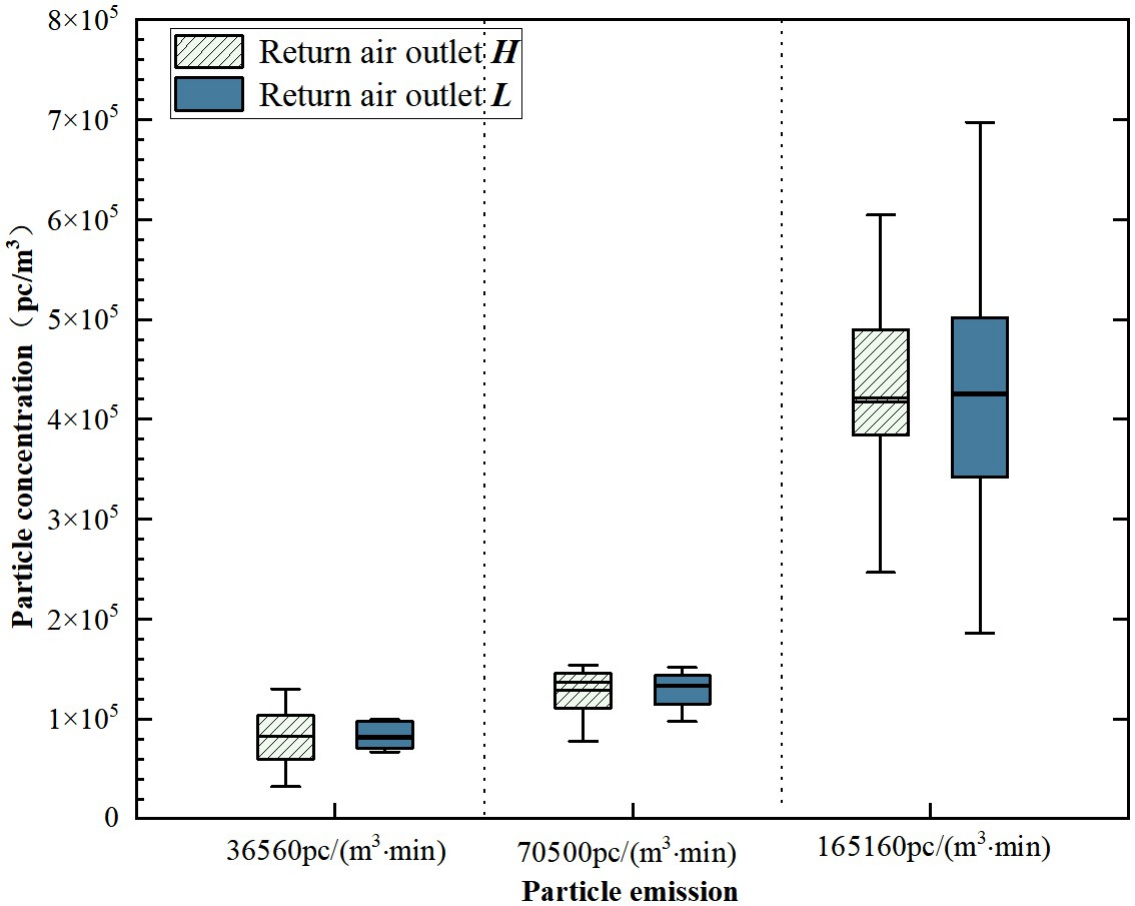
Concentration distribution in the vicinity of the source area.

**Fig 9:**
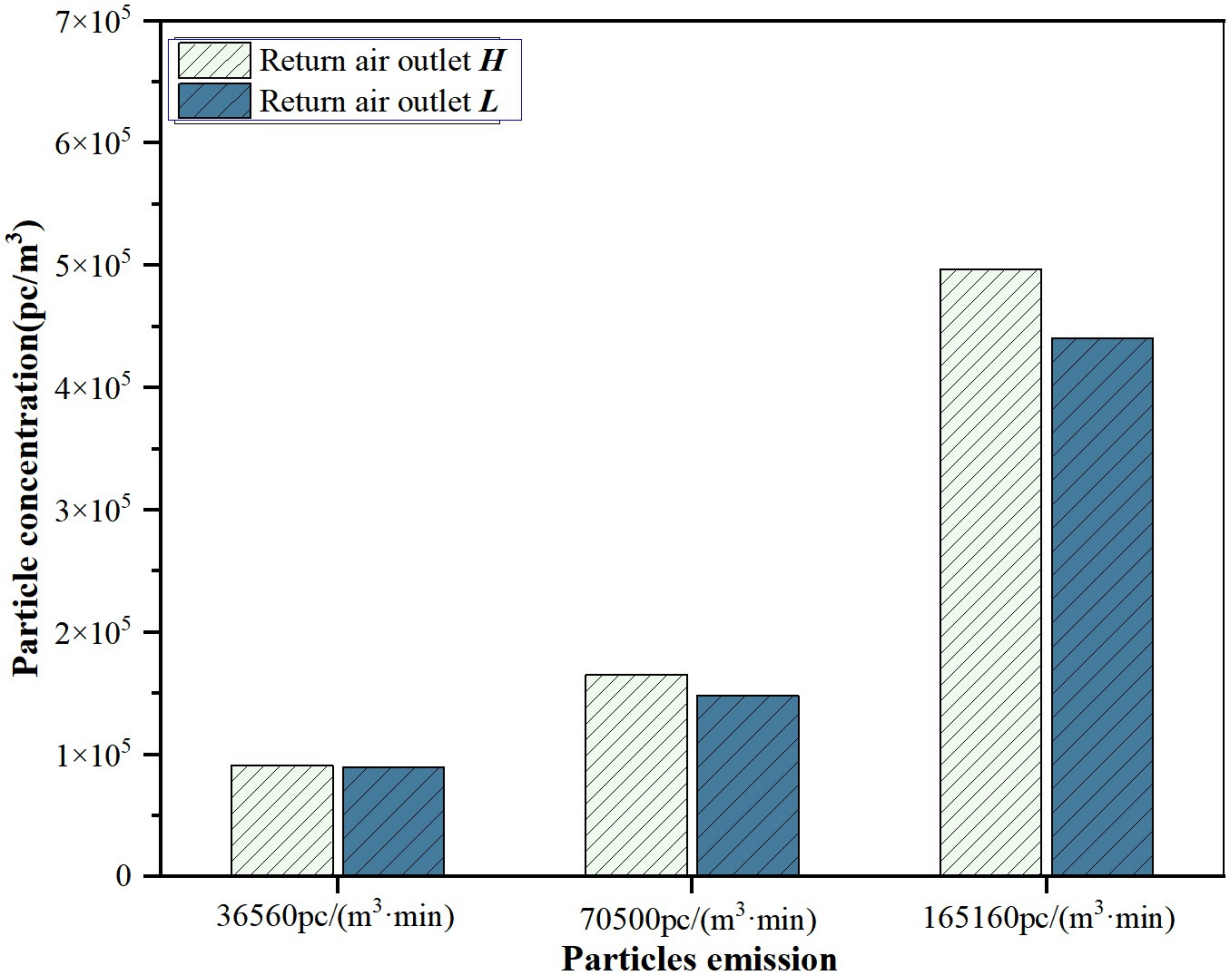
Average concentration at different return air outlet heights.

The vicinity of the emission source refers to the area near the source point, typically the active work area for individuals. 12 measuring points in the center on different heights are used to monitor and describe the concentration distribution in this region, as shown in Fig 8. In this area, the concentration values are similar between the two measurement points. Compared to return air outlet ***L***, the concentration distribution at return air outlet ***H*** is more scattered when one or two persons. With the emission of five persons, the concentration distribution at return air outlet ***H*** shows a relatively concentrated and right-skewed pattern. The average concentration of the three measurement heights was taken as the indoor concentration. Fig 9 illustrates the variation in indoor concentration. Under return air outlet heights, the indoor particulate concentration increases with an increase in the particle emission rate. For the three particle emission rates, the average concentration at return air outlet ***H*** is 2.0%, 11.4%, and 12.7% higher than the average concentration at return air outlet ***L***. The difference in average concentration is more pronounced at the particle emission rate of 165160pc/(m^3^·min). With the particle emission from a single person, both configurations show very similar concentration levels that meet the cleanliness requirements. In typical cleanrooms, where the number of occupants is strictly regulated, return air outlet ***H*** holds a slight advantage.

**Fig 10:**
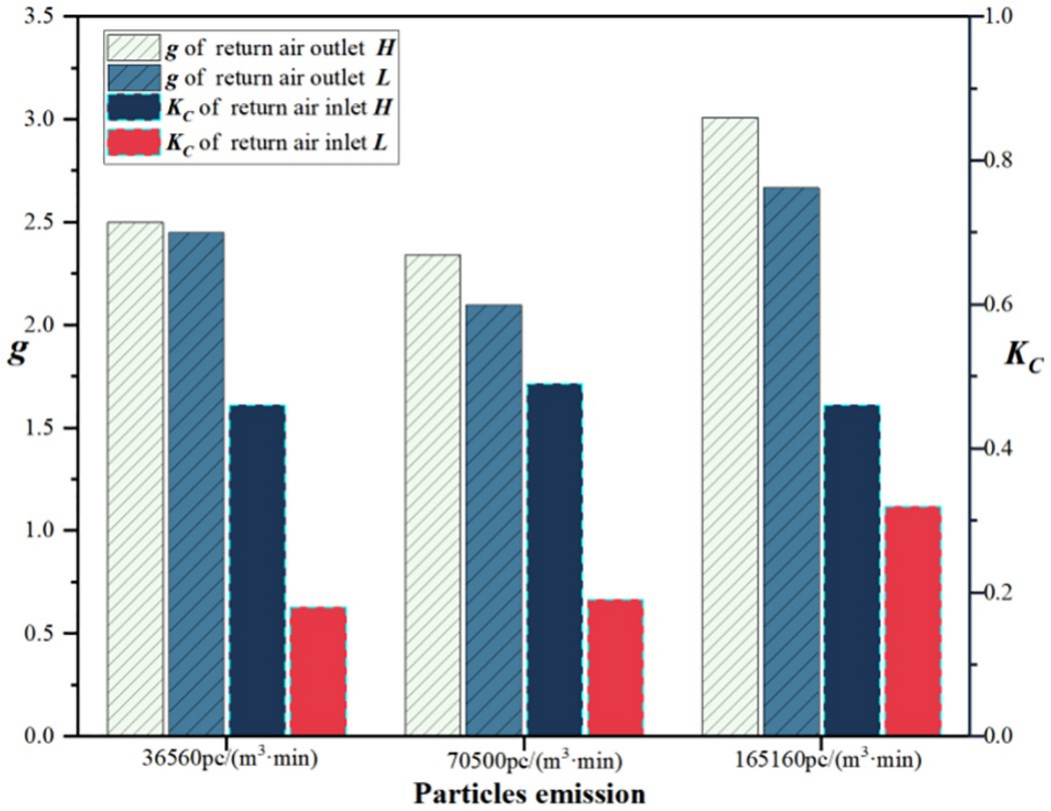
*g* and *K*_*c*_ at different return air outlet heights.

By using Eq (1), *g* is calculated for different return air outlet heights, as shown in Fig 10. *g* at return air outlet ***H*** is always higher than at return air outlet ***L***, with the largest difference observed at the particle emission rate of 165,160 pc/(m^3^·min). For both heights, *g* is lower at the particle emission rate of 70,500 pc/(m^3^·min) compared with the other emission rates, whereas it is highest at the rate of 165,160 pc/(m^3^·min), indicating that the purification capability of the airflow organization in the cleanroom is relatively weak at this emission rate. Under the same conditions, the cleanroom’s performance is slightly lower when employing return air outlet ***H***, which is not conducive to the discharge of particles. When the particle emission from a single person is only 2.1% higher, the cleanliness effectiveness of both configurations is similar.

The coefficient of non-uniformity for particle concentration is calculated using Eq (2), and the results are shown in Fig 10. As the particle emission rate increases, the coefficient of non-uniformity remains relatively stable for return air outlet ***H***, while it exhibits an increasing trend for return air outlet ***L***. Under the three particle emission rates, the coefficient of non-uniformity for return air outlet ***H*** is always higher than that for return air outlet ***L***, indicating better uniformity of indoor particle concentration when using a lower return air outlet height. This phenomenon suggests that with only one person in the room, a lower return air outlet height provides better uniformity in indoor particle concentration. As the number of occupants increases, the uniformity improves for return air outlet ***H*** while it deteriorates for return air outlet ***L***, resulting in a smaller difference in the coefficient of non-uniformity between the two heights. The reason might be the more thorough mixing of particles at higher particle emission rates and reduced influence of the return air outlet. However, it is unlikely for the room to reach a five-person occupancy, and the comparison is most significant between one and two-person particle emission rates. After an increase in particle emission, the particles mix more thoroughly, resulting in a reduced impact of the return air outlet on air uniformity.

#### 3.1.2 Influence of the width change of the return air outlet on the particle distribution

The experimental results indicate that a inferior cleanroom performance is achieved with return air outlet ***H*** oriented towards operators, particularly under high particle emission conditions. We would have used the experimental conditions with the return air height ***H*** to study the influence of the return air outlet width on the distribution of particle concentration with the most unfavorable particle emission for five persons.

**Fig 11:**
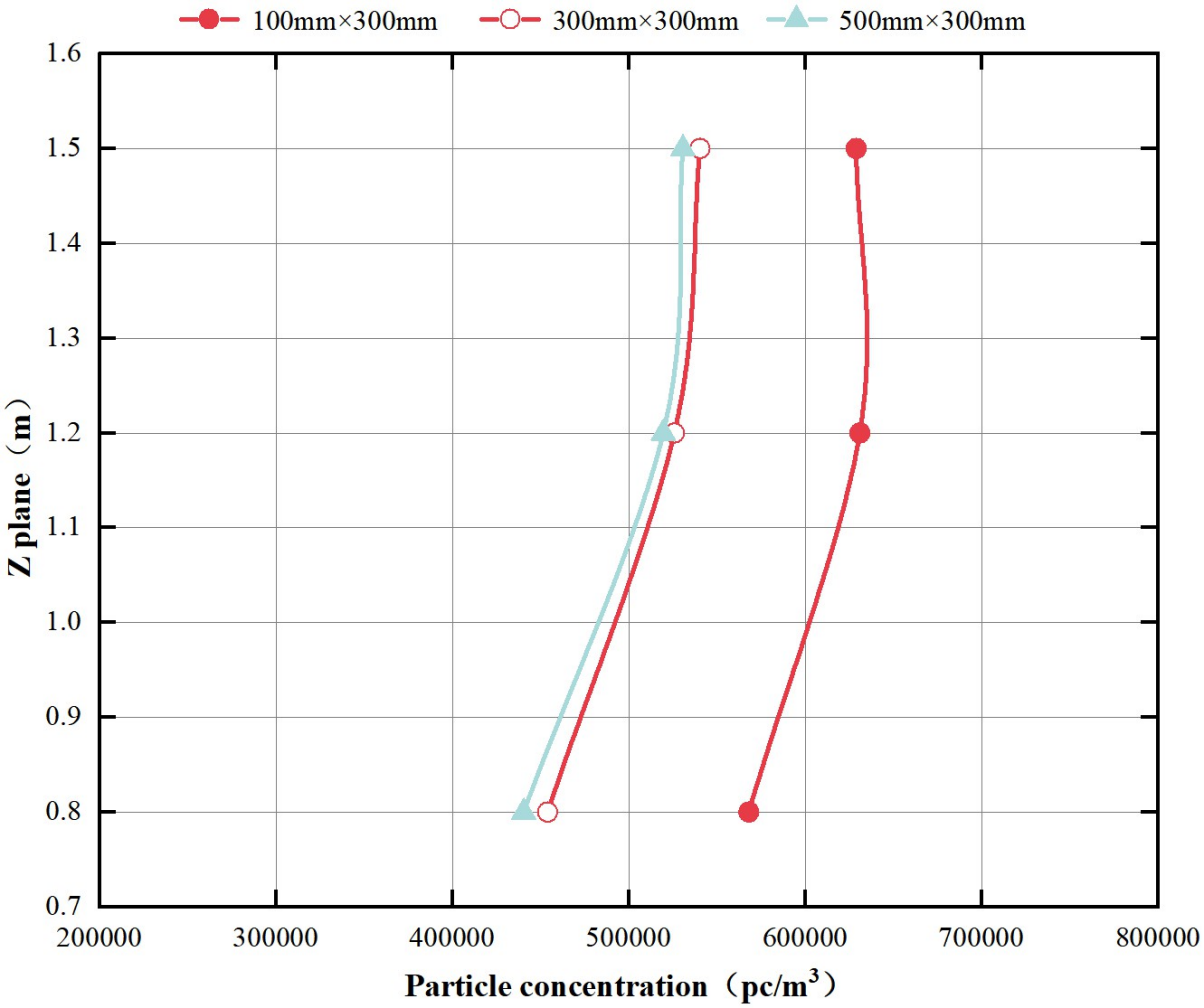
Vertical concentration at different return air outlet widths.

The vertical concentration results for different return air outlet widths are shown in Fig 11. Details of the full results are shown in S2 Table. The vertical concentration is higher for a 100 mm width return air outlet compared with the other two sizes, which may be due to the smaller size return air outlet having a higher airflow velocity, leading to increased shear stress between the layers of fluid and promoting the generation of turbulent flow. It enables particles to flow downward and reduces the likelihood of expulsion. Due to the close proximity of the return air outlet ***H*** to the emission source, the downward flow of particles is constrained to some extent. Consequently, the concentration measured at a height of 0.8m is relatively low.

**Fig 12:**
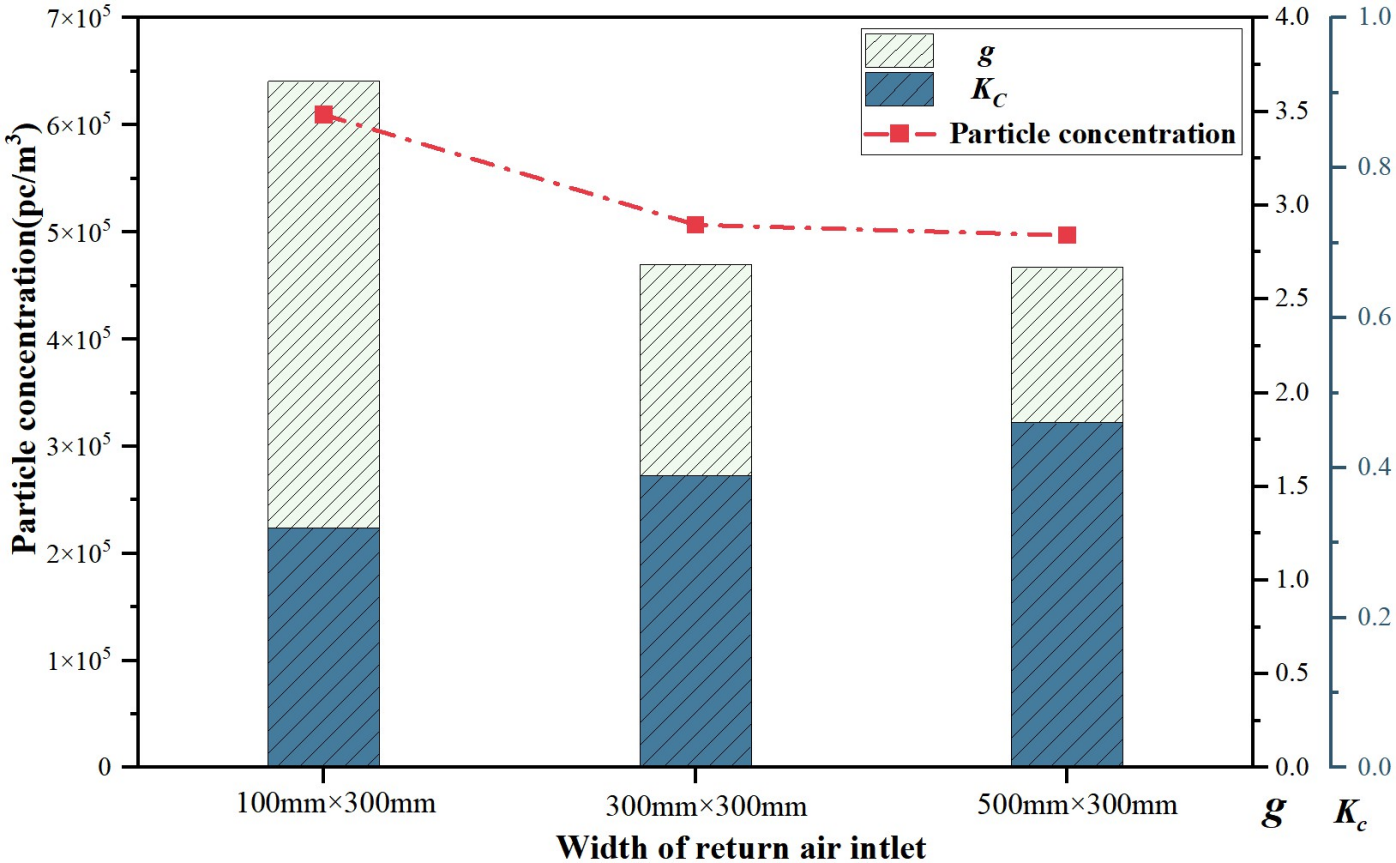
Average concentration at different return air outlet widths.

Fig 12 depicts the concentration, *g* and *K*_*c*,_ for different return air outlet widths. The average particle concentration is 16.8% and 18.5% higher for the 100 mm width return air outlet compared with the 300 and 500 mm width return air outlets, respectively. The larger return air outlet size has a wider impact area, increasing the probability of particle flow toward the vicinity of the return air outlet and facilitating particle exhaust. Thus, larger return air outlets have a favorable effect on concentration control.

With a 100 mm width return air outlet, *g* is relatively high, indicating a lesser purification effect influenced by turbulence. As the indoor return air outlet width increases to 300 mm, concentration-to-dust ratio significantly decreases, signifying a better purification effect due to improved airflow organization. However, further increasing the return air outlet width does not lead to substantial reductions in indoor particle concentration or *g*, which may be because, at the 300 and 500 mm return air outlet widths, the respective air velocities are 1.01 and 0.61 m/s, and the differences in confluence are not significant. The uniformity of particle concentration gradually decreases with an increase in width.

### 3.2 Numerical result analysis

Increasing the particle emission rate causes a decrease in the coefficient of non-uniformity for the return air height ***H***, whereas the opposite trend occurs for the return air height ***L***. Although the cleanliness level under return air outlet ***H*** is lower than that under return air outlet ***L***, these experimental results were obtained under the condition of 11 air changes per hour. However, cleanrooms of ISO Class 7 or above generally have an air exchange rate higher than 20 times per hour. In various studies, many factors influence the distribution of particle concentration in non-unidirectional flow cleanrooms, among which the air change rate and particle emission rate are the main factors.

To compare the control capability of the return air outlet ***H*** on indoor particles, the distribution of particles at different particle emission rates and air change rates under the two return air outlet heights was simulated and studied based on the theory of uniform distribution, using the calculation curve of indoor particle concentration *N* = *f*(*G*/*n*).

#### 3.2.1 Analysis of average concentration results

**Fig 13:**
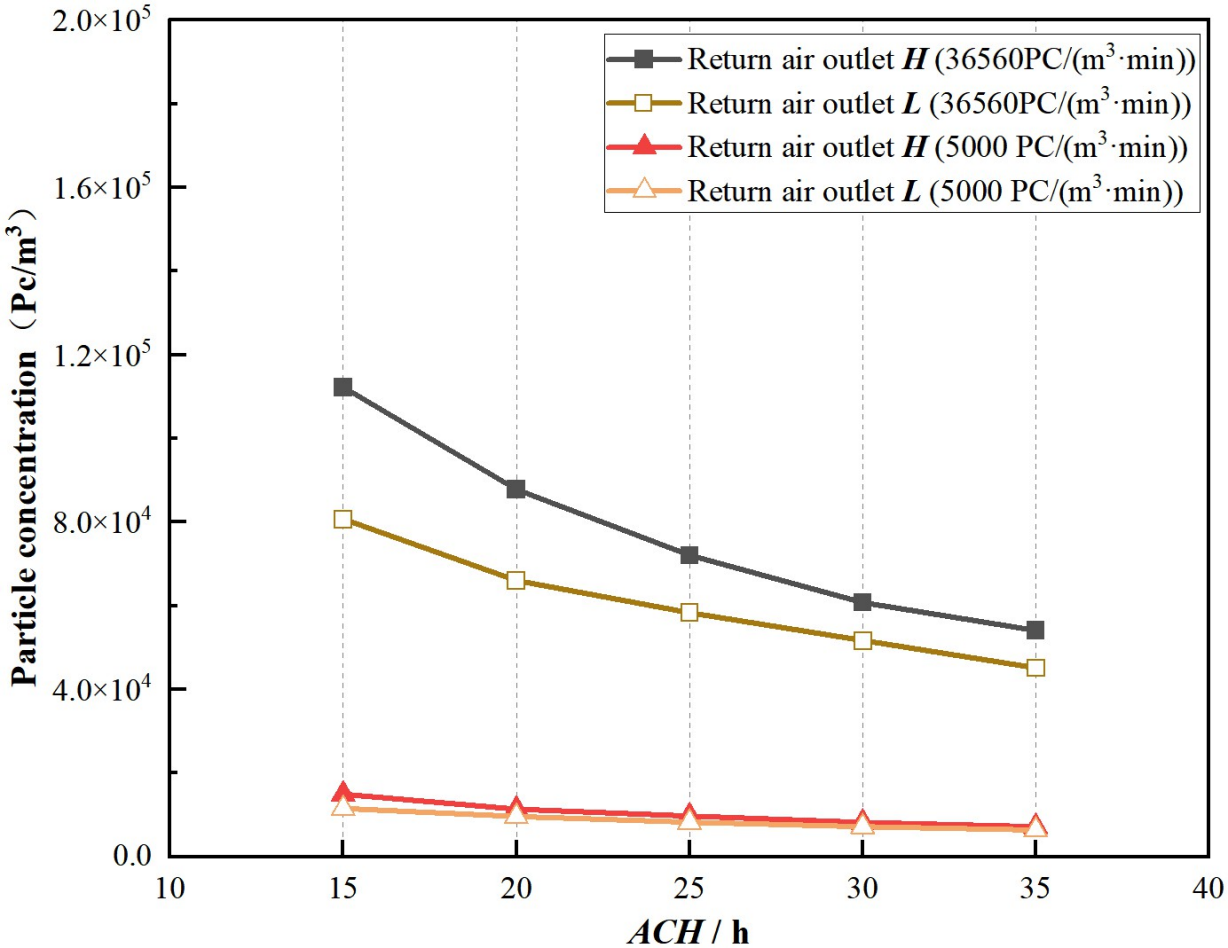
Average particle concentration at variation of ACH under two return air outlets.

Fig 13 illustrates the variation of indoor particle average concentration with air change rate for two different particle emission rates and various return air heights. Under both emission rates, as the air change rate increases, the clean air supply in the indoor environment increases, enhancing the purification capability of the cleanroom. Consequently, the concentration in the indoor environment gradually decreases in a slowing-down manner for all conditions. The average concentration for return air outlet ***H*** is consistently higher than that for return air outlet ***L***, and the gap between the two decreases as the air change rate increases. For higher emission rates, the reduction in particle concentration is more significant compared with lower emission rates. With increasing air change rate, the particle concentration for return air outlet ***H*** increases by 12.34% to 22.71% and 14.92% to 28.07% relative to the values for return air outlet ***L*** for the two emission rates. Therefore, a lower return air height can enhance the purification capability of the cleanroom, especially when the particle emission rate is higher and the effect is significant. Nevertheless, with a particle emission rate of one person or less, and the air exchange rate is 20 times per hour or higher, both types of return air outlets meet the cleanliness requirements.

FFU is responsible for supplying clean air into the room to achieve dilution and removal of pollutants. The indoor pollutant concentration can be reduced by increasing the air change rate. However, after reaching a certain air change rate, the rate of decrease in pollutant concentration in the cleanroom gradually diminishes, leading to a wastage of energy for the air supply. Lowering the return air height can improve the cleanliness level of the cleanroom to some extent. However, in situations with high particle emission rates, increasing the air change rate remains the most direct method to control particle levels.

**Fig 14:**
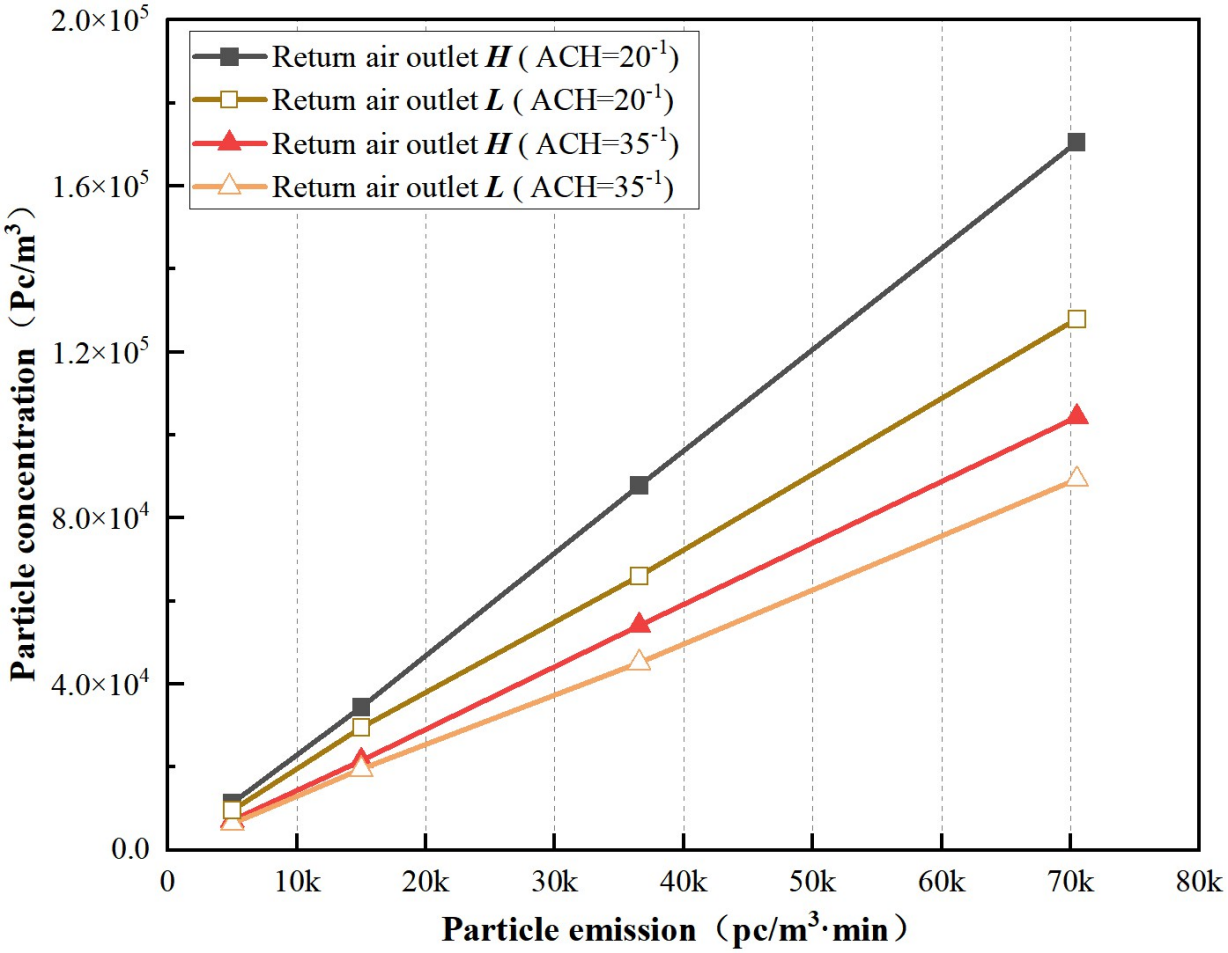
Average particle concentration at variation of particle emissions under two return air outlets.

From Fig 14, the trend of the average indoor particle concentration is consistent. As the particle emission rate increases, the average indoor particle concentration gradually increases. With an increase in air change rate, the return air outlet ***H*** increases by 17.96% to 33.23% and 12.43% to 16.81% compared to ***L***. Higher air exchange rates have a significant impact on reducing indoor particle concentration. At this point, when using the outlet height ***L***, the concentration reaches its lowest level, especially at higher particle emission rates. Compared with the particle concentration at an air exchange rate of 35/h with ***L***, the return air outlet ***H*** at an air exchange rate of 20/h is 77.54% to 90.86% higher for the four particle emission rates. Therefore, the combined effect of both factors contributes greatly to improving cleanliness. For a particle emission rate of two persons, an air exchange rate of 35 times per hour precisely satisfies the ISO Class 6 cleanliness requirements, requiring an air exchange rate higher than 35 per hour.

**Fig 15:**
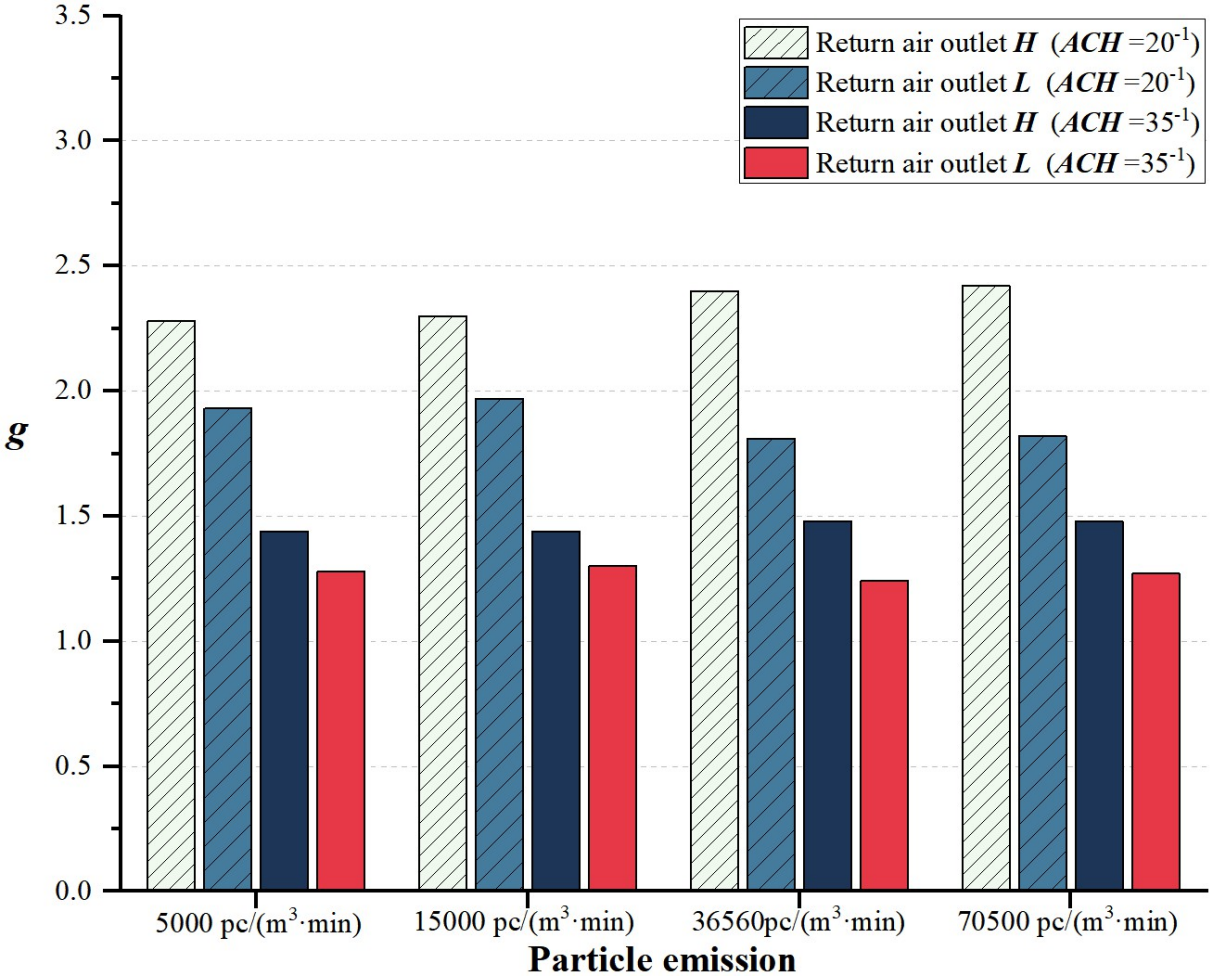
Variation of particle emissions under two return air outlets (*g*).

**Fig 16:**
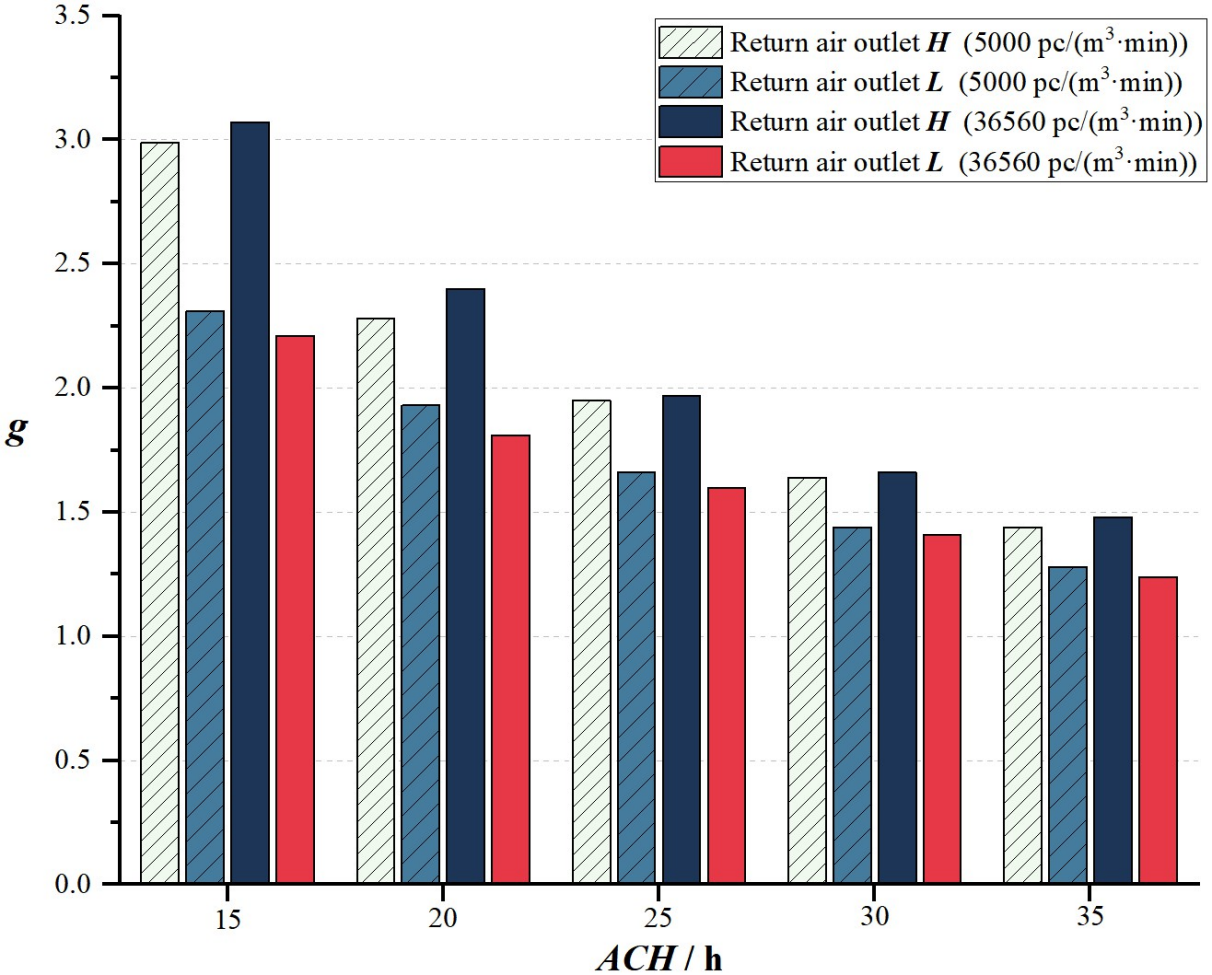
Variation of ACH under two return air outlets (*g*).

In Fig 15, for all conditions, *g* for the return air outlet ***L*** is lower than ***H***. When the air change rate is kept constant, slight variation is noticed in the particle emission rate with return air outlet ***H***. Among the two air change rates, higher air change rates have better purification effects in the indoor environment. Under the same air change rate, the cleanroom purification capability is stronger for return air outlet ***L*** compared with ***H***. Fig 16 demonstrates that, for a fixed particle emission rate, *g* decreases with an increase in the air change rate, and the difference between the return air outlet ***H*** and ***L*** gradually decreases. Under high-clean air flow conditions, the advantage of the airflow path for the return air outlet ***L*** reduces. With an air exchange rate of 30 and 35 times per hour, the variation in the concentration of particles is within 15%. In summary, under the same conditions, a higher air change rate and installing the return air outlet ***L*** result in a lower concentration to dust ratio, indicating that the return air outlet ***L*** achieves better purification effects compared to ***H***.

#### 3.2.2 Analysis of concentration uniformity results

**Fig 17:**
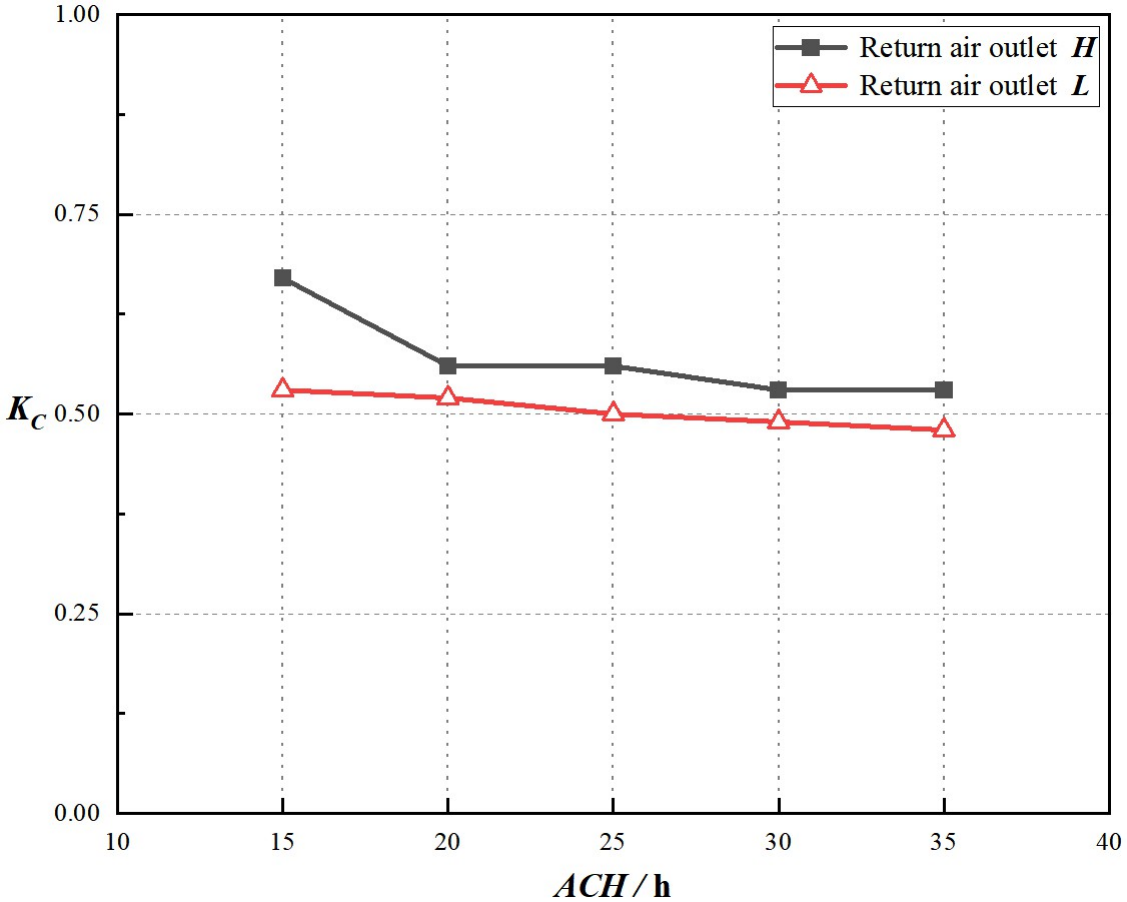
*K*_*c*_ at the particle emission rate = 5000pc/(m^3^·min).

**Fig 18:**
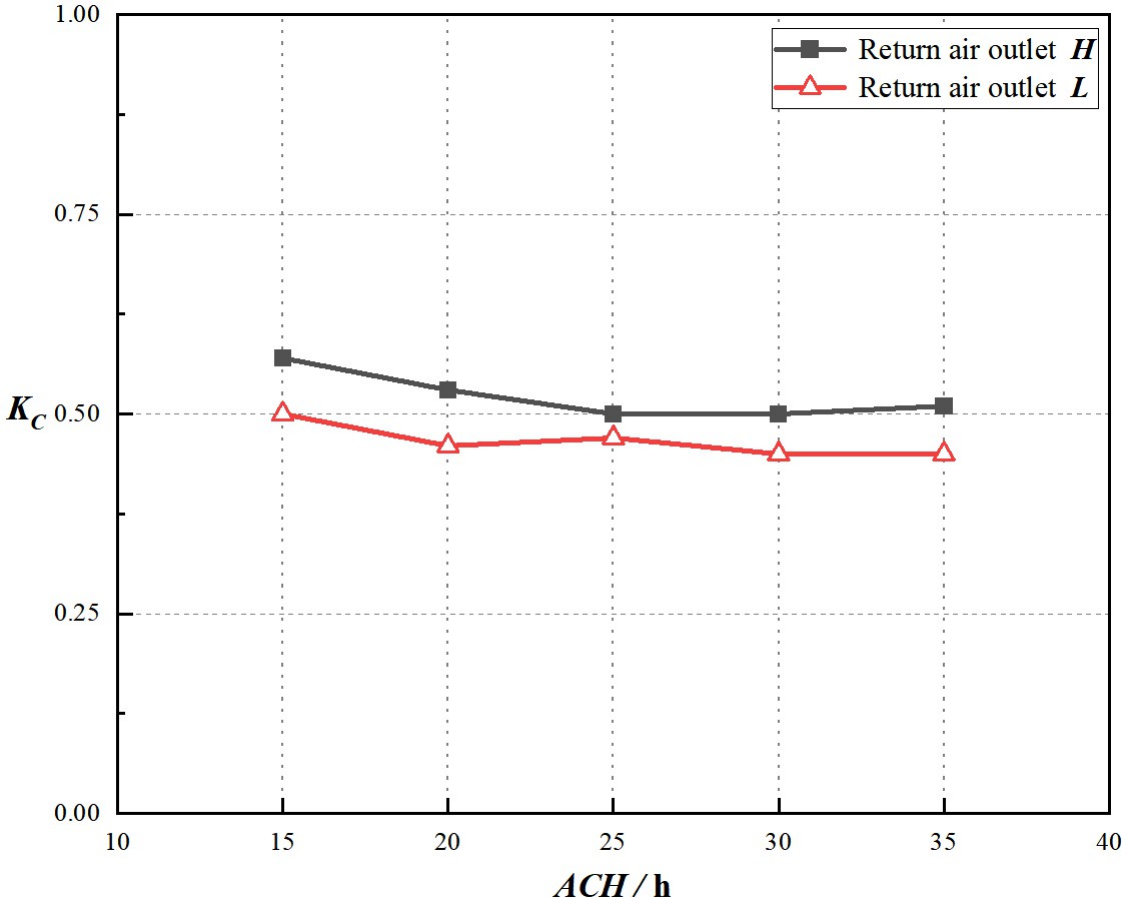
*K*_*c*_ at the particle emission rate = 36560pc/(m^3^·min).

Using Eq (2), the calculation of the coefficient of non-uniformity for indoor particle concentration at different air change rates is shown in Figs 17 and 18. For each air change rate, the indoor particle uniformity for return air outlet ***L*** is superior to that for return air outlet ***H***. Moreover, when the particle emission rate is 5000 pc/(m3·min), the average difference in particle concentration non-uniformity coefficient between the two heights is the largest, approximately 0.07. As the air change rate increases, air diffusion becomes faster, resulting in better dilution effects and a more uniform distribution of particle concentration. In all simulated conditions, both for return air outlet ***H*** and ***L***, the average non-uniformity coefficient is lower for higher particle emission rates, indicating better uniformity. It is approximately 0.92 times that of the low particle emission rate conditions.

**Fig 19:**
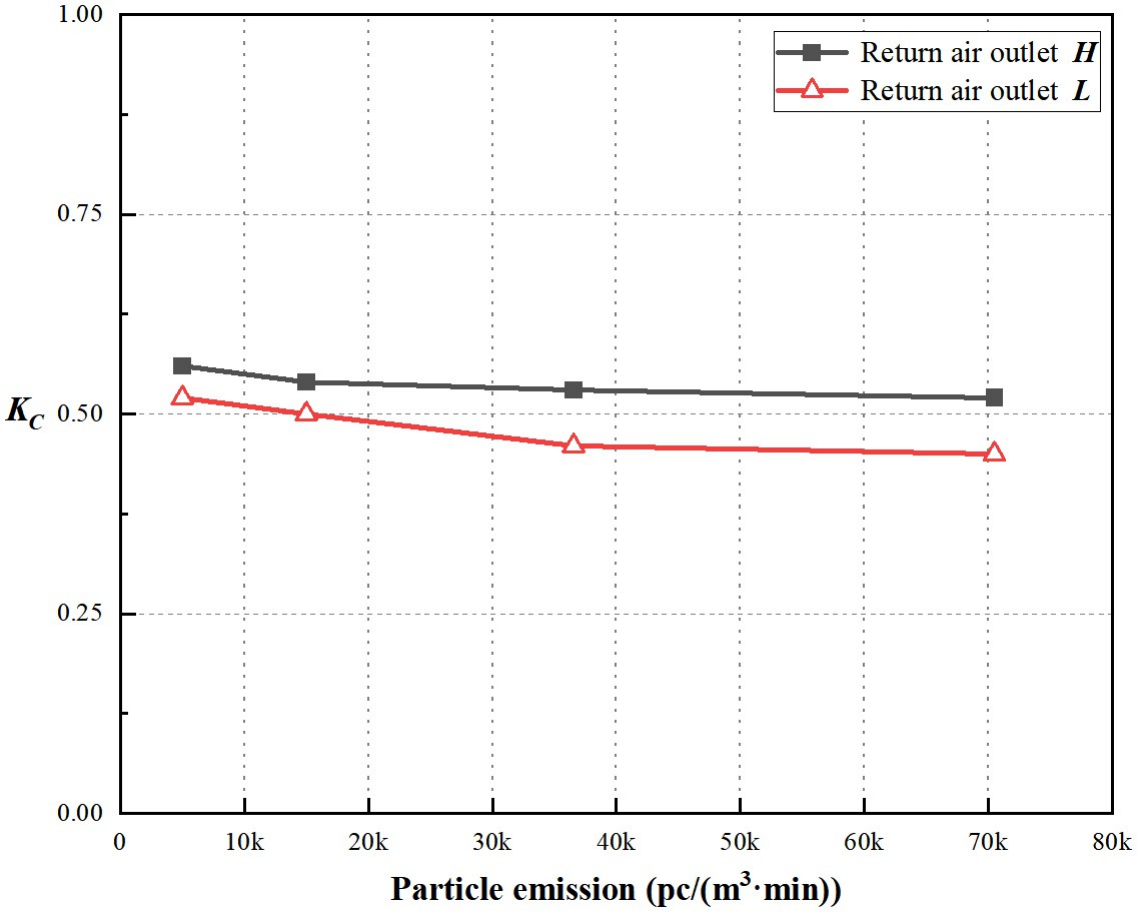
*K*_*c*_ at ACH = 20/h.

**Fig 20:**
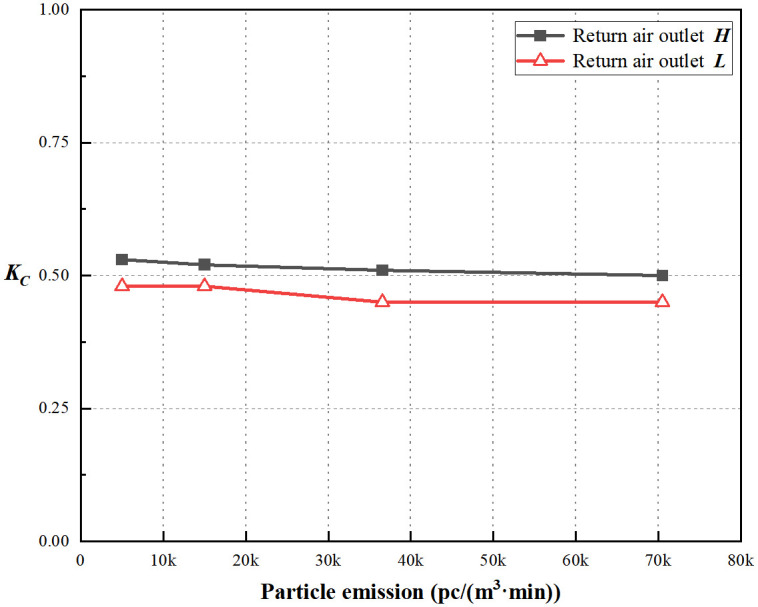
*K*_*c*_ at ACH = 35/h.

Based on Figs 19 and 20, the coefficient of non-uniformity for particle concentration decreases with an increase in the particle emission rate for both return air outlets ***H*** and ***L***, indicating that, in this simulated condition, a higher particle emission rate leads to a more uniform distribution of particle concentration. More details of these results can be found in S3 Table. Under both air change rates, for the same return air height, the average coefficient of non-uniformity for indoor particle concentration is approximately 0.96 times that of the lower air change rate. Such, higher air change rates result in better uniformity distribution of particle concentration. This observation reveals that the uniformity of the return air outlet ***H*** is not significantly compromised with air change rates of 20 times per hour or higher.

### 3.3 Calculation of the difference in air supply volume under different return air heights

According to Eq (3), when the cleanroom’s supply air is in a steady state, the indoor stable particle concentration *N* is related to the particle emission rate *G* and the air change rate *n*, i.e., *N* = *f*(*G*/*n*). Based on the relationship between *N* and the particle emission rate *G* and air change rate *n*, the theoretical calculation curve can be plotted. Using this curve, the particle concentration (i.e., cleanliness) can be calculated for different air change rates while keeping the indoor particle emission rate constant. When the indoor particle concentration (cleanliness level) and the particle emission rate are known, this curve can be used to deduce the corresponding air supply volume.

**Fig 21:**
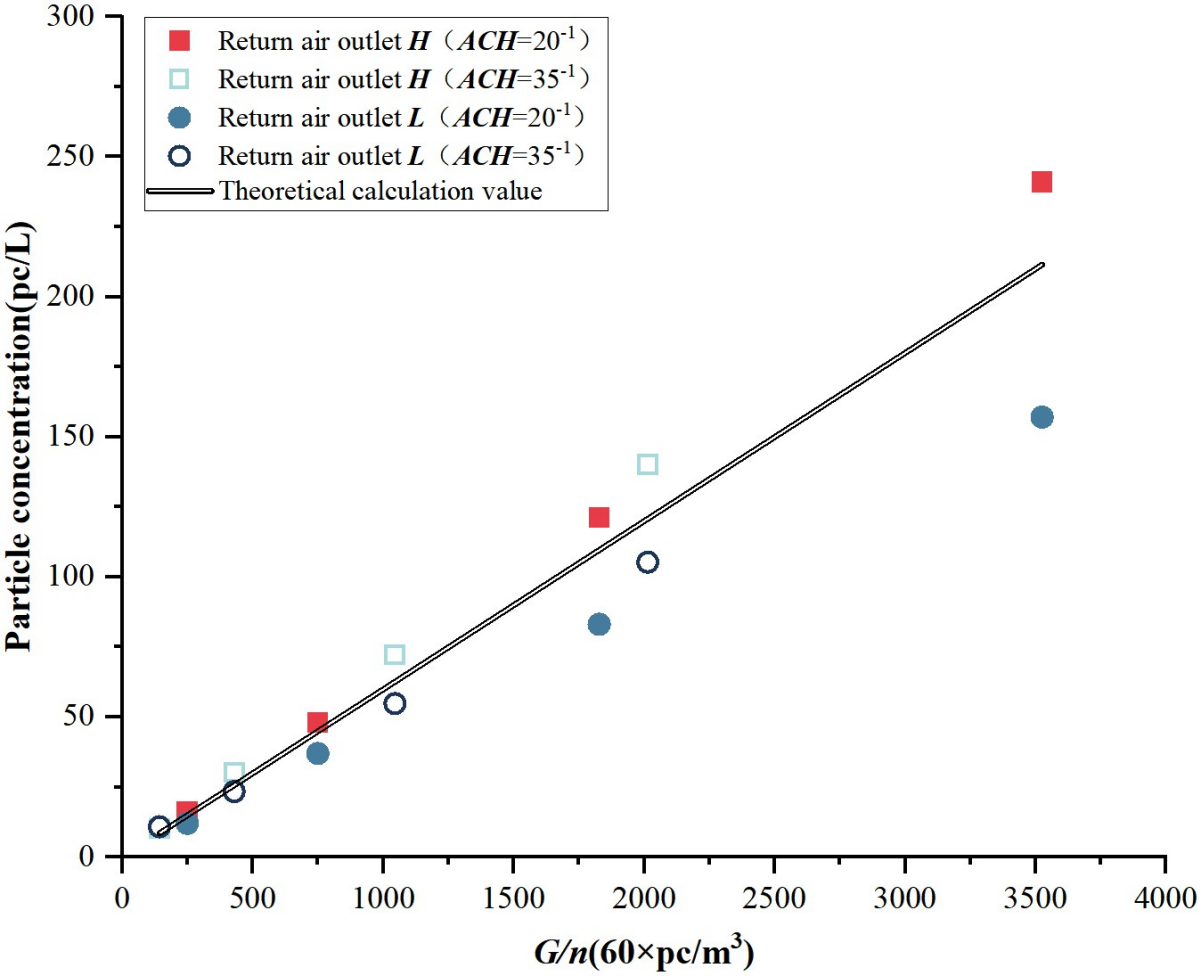
The theoretical and simulated comparison of concentration for a supply air outlet size of 400mm × 400mm.

Fig 21 depicts a comparison between the theoretical calculation curve and the variation of indoor particle concentration with particle emission rate for different return air heights under two air change rates, assuming a supply air outlet size of 400 mm × 400 mm and a return air outlet size of 500 mm × 300 mm. When the return air height is ***H***, the numerically simulated particle concentrations are higher than the values obtained from the theoretical calculations for both air change rates. Furthermore, this difference increases as *G*/*n* increases. Conversely, when the return air height is ***L***, the simulated results yield lower particle concentrations compared with the values obtained from theoretical calculations.

**Fig 22:**
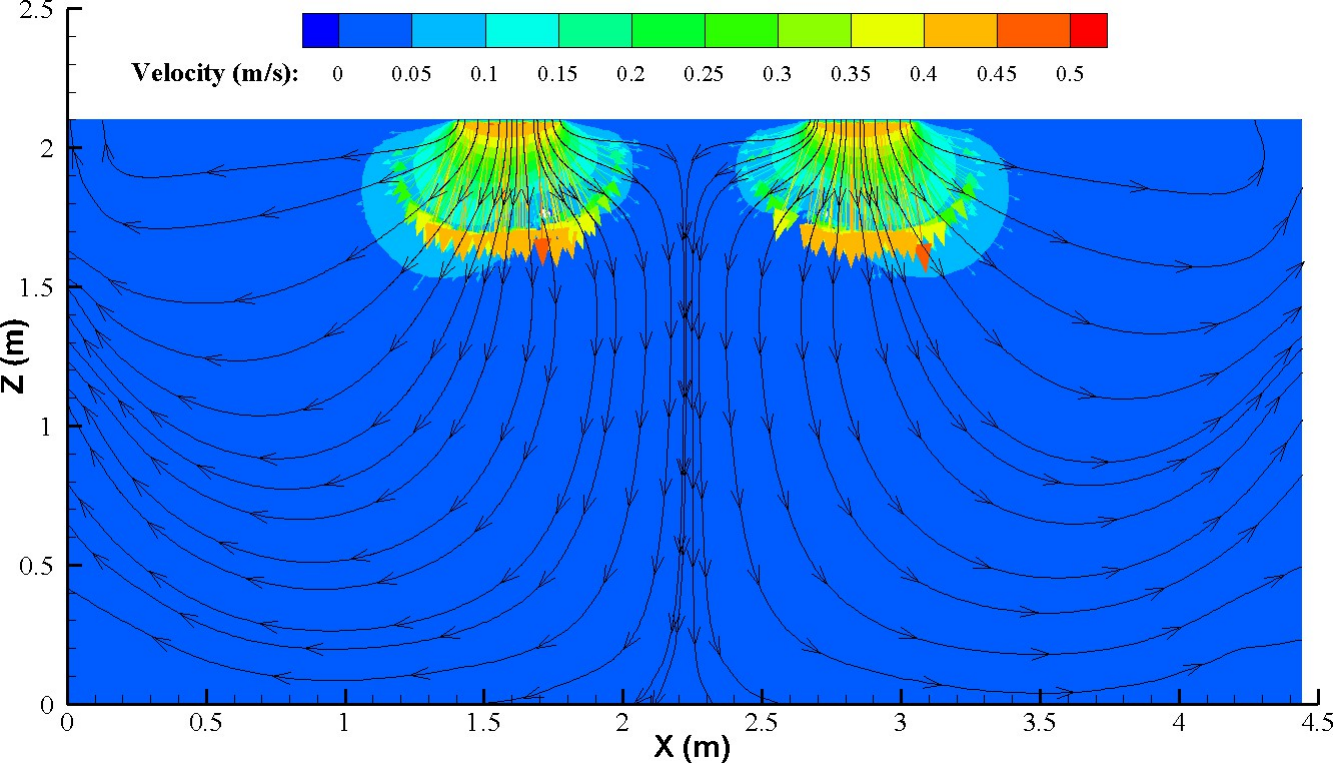
The vector diagram of the flow field and the streamline (ACH = 30/h).

The observed phenomenon may be due to the following reasons: When the return air height is ***H***, as the particle emission rate increases, the content of indoor particles retained in the turbulent flow zone increases. Moreover, since the return air outlet is close to the supply air outlet, in this case, particles at the return air outlet are more prone to "short-circuiting," meaning they cannot be promptly expelled from the room, resulting in higher indoor particle concentrations. On the other hand, when the return air height is ***L***, it is located further away from the supply air outlet, allowing for a more thorough mixing of indoor particles with the airflow from the FFU units. Furthermore, the greater distance between the return air outlet and the supply air outlet enables faster dilution and timely expulsion of particles from the turbulent flow zone, resulting in lower concentrations compared to the values obtained from theoretical calculations. However, based on the observation of the vector diagram of the flow field and the streamline(Fig 22), it can be concluded that there is no complete short-circuiting phenomenon present at increased air change rates. In the vicinity of the return air outlet, the higher velocity airflow is largely unaffected by the return air. The majority of the airflow is directed in a vertical downward motion, with lower airflow intensity observed near the ground level. Nevertheless, the lower return air is prone to be influenced by the indoor layout, which can obstruct the removal of particles.

To compare the simulation results with the theoretical calculations, a coefficient called the airflow coefficient *ξ* is calculated, which represents the difference between the simulated indoor particle concentration *N*_*model*_ and the theoretically calculated values *N*_*t*_. The formula for calculating the airflow coefficient is given by Eq (4).

$$\xi =\frac{N_{model}}{N_{t}},$$ (4)

where *N*_*model*_ represents the indoor particle concentration obtained from a specific operating condition in the simulation. It can be obtained by taking the average of the concentration values at various points in the simulated indoor environment. On the other hand, *N*_*t*_ represents the theoretically calculated indoor particle concentration for that operating condition.

The previous analysis suggests that airflow organization and particle distribution exhibit certain non-uniformity in the cleanroom. Consequently, the particle concentration in the cleanroom is also non-uniform distribution. *ζ*_*t*_ is closely related to the non-uniformity of particles. To describe the influence of airflow non-uniformity under different conditions quantitatively, the *N-n* equation from Professor Xu’s "Fundamentals of Air Cleaning Technology and Its Application in Cleanrooms" [39] can be applied for analysis.

$$N=\xi_{t}\frac{60G\times 10^{-3}}{n},$$ (5)

where ξ is primarily related to airflow uniformity and the location of indoor particle sources. The height of the indoor particle emission source is approximately 1.7 m. When the return air outlet is at height ***L***, the particle source is located between the path from the supply air outlet to the return air outlet. Therefore, particles can be considered to be mainly distributed in the mainstream region. However, in the case of the return air outlet ***H***, where the height is slightly lower than the particle source, the supply airflow reaches the particle source to dilute the particles, and the particles experience inertial forces and tend to enter the turbulent flow zone of the room. Therefore, this airflow organization has an increased likelihood of particles being retained in the clean space, leading to an overall increase in indoor particles. The placement of the return air outlet ***L*** may be influenced by the layout of processes, leading to disturbances in the airflow path and increased particle accumulation. On the contrary, placing the return air outlet ***H*** on the upper side not only addresses this issue but also greatly enhances space utilization.

According to Fig 16, for the same indoor particle concentration, the *G*/*n* value at the return air outlet ***L*** is larger than the return air outlet ***H***, which indicates an increased allowable particle emission rate when the same air change rate. Similarly, for the same particle emission rate *G*, the air change rate ***n*** at the return air outlet ***L*** can be smaller than the air change rate ***n*** at the return air outlet ***H***. Under the same cleanliness level, *ξ* also represents a variation in the supply air volume relative to the theoretical calculation, $\xi =\frac{n_{i}}{n_{t}}$. *n*_*i*_ refers to the air change rate in the target space, and *n*_*t*_ refers to the air change rate in the theoretically calculated. *η* represents the variation of relative change.


$$\begin{array}{l} \eta =\left(\frac{n_{i}}{n_{t}}-1\right)\times 100\% \end{array}$$ (6)

For the same indoor particle concentration standard, the value of *G*/*n* at the height H of the return air outlet is smaller compared to the height ***L***. When the supply air outlet has dimensions of 400 mm × 400 mm and the return air outlet have dimensions of 500 mm × 300 mm, assuming the particle emission rate *G* and indoor particle concentration *N* have the same values at heights ***H*** and ***L***, the air change rate required at return air outlet ***H*** is approximately 1.22 times that ***L***. Similarly, the airflow coefficients can be calculated for different supply air outlet sizes, as shown in Table 3. The airflow coefficient at height ***H*** is higher than that at height L. Therefore, to achieve the same cleanliness level, the supply air quantity required at height ***H*** is 11% to 25% higher than that required at height ***L***. With a common supply air outlet size of 600mm in most cleanrooms, incorporating return air outlet ***H*** only necessitates an additional 11% air volume to meet the cleanroom standards.

**Table 3:** Airflow coefficients.

supply air outlet sizes (mm×mm)	Return air outlet *H*		Return air outlet *L*	
	*ξ*	*η*	*ξ*	*η*
300×300	1.15	+15%	0.90	-10%
400×400	1.13	+13%	0.93	-7%
500×500	1.12	+12%	0.90	-10%
600×600	1.09	+9%	0.98	-2%

## 4 Discussion

This research provides particle concentration and airflow distributions under return air outlet ***H*** and shows the value of its applicability as compared to a conventional lower-side return air outlet. In cleanrooms, the traditional lower-side return air outlet may be affected by equipment arrangement and operators activities, leading to particle retention. In contrast, return air outlets oriented towards operators can not only improve space utilization but also provide less impact from upper sources on the workbench area. In some specific clean environments, such as operating rooms in hospitals, using higher-side return air outlets can reduce the risk of secondary infection and control particle concentration near the workbench [33, 35].

Return air outlet ***H*** is suitable for situations with complex process equipment to increase space utilization and avoid contamination of clean rooms below the workbench height. However, in cases where the equipment layout near the return air is singular and only cleanliness is required, the effectiveness of the return air outlet ***H*** is not as well as that of the return air outlet ***L***. We have not analyzed the flow field under the dynamic influence of various process equipment or personnel near the return air outlet L in scenarios. Therefore, from the perspective of design, using return air outlet ***H*** without considering other factors may lead to unstable effectiveness. This research determines the particle concentration, uniformity, and required supply air volume for return air outlet ***H*** and ***L*** under various conditions, providing guidance for further research on higher-side return air outlet. It extends beyond traditional clean room return air design. Some studies [27, 32, 35, 37] also indicates constraints in certain situations with traditional low return air, confirming the potential mentioned above. Subsequent research can be expanded and verified through experiments.

## 5 Conclusion

This article utilizes experimental and simulation approaches to investigate the impact of return air outlet oriented towards operators as particle emission sources on particle concentration distribution in non-unidirectional flow cleanrooms. A comparison is made with traditional lower-side return air outlets, and a method combining uniformity theory is provided for calculating the air supply volume differences. Through experimentation, the study reveals that the purification efficiency and uniformity of particle concentration at the return air outlet ***H*** slightly decrease compared to the conventional lower-side return air outlets. Under the influence of particle emission, the particle concentration increases by 2.0%, 11.4%, and 12.7%. When the particle emission rate is equivalent to that of one person, the concentration values are very close.

Based on the experimental results, simulations are conducted to further determine the applicability of return air outlet ***H***. Under the same conditions, the particle concentration with return air outlets ***H*** increases by 12.34% to 33.2% compared to return air outlet ***L***, and the concentration difference between the two decreases as the air change rate increases. For different levels of particle emission inside the room, 20 ACH is sufficient to meet the ISO Class 6 cleanroom requirements with return air outlet ***H*** when there is one person in the room, while a rate of 35 ACH is needed for two persons. The ranges of the non-uniformity coefficients for return air outlet ***H*** and ***L*** are 0.50 to 0.67 and 0.45 to 0.53, respectively. The average non-uniformity coefficient of return air outlet H higher 11.9% than return air outlet L. These findings indicate that return air outlet ***H*** also possess a certain level of cleanroom capability, with minimal variation in uniformity compared to return air outlets ***L***. They can be applicable in cleanrooms with complex equipment. While return air outlet ***L*** have some potential in reducing particle concentration in the cleanroom, increasing the air change rate remains the most direct method of controlling particle concentration. With a common supply air outlet size of 600mm×600mm, return air outlet ***H*** only require an additional 11% of air volume to meet the same cleanroom requirements.

## Supporting information

S1 Table The model validation. Contains all validation results for the correctness of the turbulence model.
(XLSX)

S2 Table Experimental major data. Contains measurement data and results from experiments performed.
(XLSX)

S3 Table Simulation major data. Contains full results of the simulation.
(XLSX)
